# Prognostic Value of UBE2S, HIF‐1α, and FOXM1 Expression in Esophageal Squamous Cell Carcinoma

**DOI:** 10.1155/ancp/3557238

**Published:** 2025-12-20

**Authors:** Wan Li, Keming Zhou, Mengyan Li, Liping Su, Xianwen Liu, Yuanyuan Lv, Yuqing Ma

**Affiliations:** ^1^ Department of Pathology, The First Affiliated Hospital, Xinjiang Medical University, State Key Laboratory of Pathogenesis, Prevention and Treatment of High Incidence Diseases in Central Asia, Urumqi, Xinjiang, China, xjmu.edu.cn; ^2^ Department of Hypertension Center, People’s Hospital of Xinjiang Uygur Autonomous Region, Xinjiang Hypertension Institute, Key Laboratory of Xinjiang Uygur Autonomous Region Hypertension Research Laboratory, NHC Key Laboratory of Hypertension Clinical Research, Xinjiang Clinical Medical Research Center for Hypertension (Cardio-Cerebrovascular) Diseases, Urumqi, Xinjiang, China, xjrmyy.com

**Keywords:** esophageal intraepithelial neoplasia, esophageal squamous cell carcinoma, FOXM1, HIF-1α, prognosis, UBE2S

## Abstract

**Objective:**

The present investigation was aimed to examine ubiquitin‐conjugating enzyme E2S (UBE2S), hypoxia‐inducible factor (HIF)‐1α, and factor forkhead box M1 (FOXM1) levels in esophageal squamous cell carcinoma (ESCC) and esophageal low‐grade intraepithelial neoplasia (LIN), high‐grade intraepithelial neoplasia (HIN). Additionally, the investigation explored the correlation between these levels and clinicopathological features in addition to prognosis.

**Methods:**

To investigate the expression patterns of UBE2S, HIF‐1α, and FOXM1, immunohistochemical staining was performed on tissue samples, including LIN, HIN, ESCC, and healthy controls. Subsequently, the chi‐square test was applied to analyze the correlations between the expression levels of the three proteins and the clinicopathological features of ESCC. The impact of UBE2S, HIF‐1α, and FOXM1 on ESCC prognosis was evaluated using survival analysis. Spearman correlation analysis was employed to analyze the correlation of three proteins in ESCC, HIN, and LIN, respectively.

**Results:**

UBE2S overexpression was related to ethnicity (*p* < 0.001) and the place of the tumor (*p* = 0.021). Overexpression of HIF‐1α (*p* = 0.017) and FOXM1 (*p* = 0.015) was connected to metastasis of the lymph node. Individuals with elevated levels of UBE2S, HIF‐1α, and FOXM1 had reduced overall survival (OS) and progression‐free survival (PFS).

**Conclusion:**

The expression levels of UBE2S, HIF‐1α, and FOXM1 are closely associated with the incidence and progression of ESCC. UBE2S, HIF‐1α, and FOXM1 have potential as prognostic indicators for ESCC.

## 1. Introduction

Esophageal carcinoma is a prevalent kind of malignant illness inside the digestive system worldwide. In China, it ranks as the fourth leading reason for mortality connected with cancer. Approximately 320,000 new instances of esophageal cancer are reported, with a death rate of 30.1 per 10,000 individuals [1]. Squamous cell carcinoma and adenocarcinoma are regarded as the main common types of malignancy in the esophagus, and the former is dominant in domestic. The distribution of esophageal cancer is associated with region, and Xinjiang Province in China has a higher incidence [2]. Esophageal squamous cell carcinoma (ESCC) has a high incidence, strong invasiveness, and limited means of diagnosis and management, resulting in poor prognosis; the 5‐year survival rate for individuals with malignancy of the esophagus is 15%–20% [3]. Consequently, to treat esophageal cancer, it is significant to find the genes related to disease occurrence/development and study the mechanism for the production of targeted drugs.

Our team has studied the molecular mechanisms related to hypoxia and metabolomics for the occurrence and development of ESCC. Hypoxia‐inducible factor (HIF)‐1α is regulated by the ubiquitin‐conjugating enzyme E2S (UBE2S) to enhance the growth, invasion, and metastasis of the tumor in ESCC [4]. Nevertheless, the relationship between HIF‐1α regulation by UBE2S and the development of ESCC, as well as the related molecular mechanisms, remains largely elusive. The ubiquitin–proteasome system (UPS), regulated by E1 ubiquitin‐activating enzymes, E2 ubiquitin‐conjugating enzymes (E2s), and E3 ubiquitin ligases, governs key aspects of biological processes in eukaryotic cells through posttranslational modification of proteins [5]. UBE2S is a family of E2 proteins [6]. UBE2S modulates the degradation of proteins by the 26S proteasome and facilitates the completion of cell division, performing crucial functions in this process [7, 8]. UBE2S is highly expressed in several kinds of human cancers and is connected with the advancement of malignancy and worse prognosis in cancer of the ovary, prostate cancer, and glioma, among others. It means that UBE2S may have a crucial function in the progression of tumors [9–11]. Moreover, there is substantial proof indicating that UBE2S expression facilitates the process of epithelial–mesenchymal transition (EMT) in malignancy cells of the pancreas. This is achieved through the interaction between UBE2S and Von Hippel–Lindau (VHL) via the UPS. These findings demonstrate that UBE2S plays a crucial role in enhancing VHL/HIF‐1α/signal transducer and activator of transcription 3 (STAT3)‐induced EMT and metastasis both in vitro and in vivo by reducing the promoter activity [12].

In their investigation, Zhang et al. [13] discovered that hypoxia triggers autophagy and suppressing autophagy might suppress the metastasis of tumors and the process of EMT in malignancies of the colorectal. Overexpression of HIF‐1α has been detected in numerous types of human cancers, and this protein is linked to malignant progression and poor prognosis in cancers such as small cell lung cancer [14], ovarian cancer [15], and tongue squamous cell carcinoma [16]. This observation indicates that HIF‐1α may serve a vital function in tumor development and progression. Prior research has revealed that UBE2S binds to pVHL and mediates its degradation via the ubiquitin–proteasome pathway. Such degradation leads to the accumulation and stabilization of HIF‐1α, thereby enhancing the growth, invasive capacity, and metastatic potential of tumor cells [17]. UBE2S may have a significant influence on cervical cancer invasion and metastasis by the pathway of pVHL‐HIF [18]. Moreover, studies have shown that the UBE2S–HIF‐1α signaling pathway can serve as a predictor for invasion, metastasis, and poor prognosis in patients with ESCC [4].

The mammalian transcription factor forkhead box M1 (FOXM1) belongs to the forkhead protein family and primarily investigates cell growth at the level of somatic transcription [19, 20]. Besides promoting cell growth, FOXM1 also plays an essential function in modulating the cell cycle, promoting angiogenesis, facilitating the invasion and metastasis of malignant cells, and EMT [21]. Consequently, FOXM1 is an essential factor in the start, promotion, and progression of malignancy [22]. Recent investigations have shown that the FOXM1 gene expression is upregulated in liver cancer, prostate cancer, colorectal cancer, et cetera [23–25]. It has also been reported in ESCC that FOXM1 is significantly elevated in lesion tissue samples of individuals with ESCC and inhibition of FOXM1 can inhibit tumor progression; thus, it can be utilized as a selective target for treatment [22]. Furthermore, one investigation revealed that FOXM1’s capacity to regulate HIF‐1α–driven transcriptional activity mediates hypoxia‐triggered EMT in prostate malignancy [24].

The goal of this investigation was to assess the UBE2S, HIF‐1α, and FOXM1 levels in ESCC and examine their relationship with clinicopathological characteristics. A comprehensive investigation was conducted to determine the impact of UBE2S, HIF‐1α, and FOXM1 on the prognosis of carcinoma cells of the esophagus. The connection between HIF‐1α, UBE2S, and FOXM1 was examined by employing Spearman correlation analysis. Our objective was to discover specific genes that suppress ESCC growth in order to promote the development of specific medications for treating cancer of the esophagus.

## 2. Materials and Methods

### 2.1. Patients and Tissue Samples

Between January 2008 and December 2018, there were an overall of 173 instances of ESCC. By examining hematoxylin and eosin (H&E)–stained sections, specific paraffin‐embedded tissue blocks with tumor tissue, and the associated normal control tissue specimens were identified. These specimens were then transformed into tissue chips. The criteria for inclusion were as follows: individuals did not receive any treatment prior to surgery; the primary tumor was situated in the esophagus and not metastatic cancer from other locations; there were no additional complications or malignancies in other organs; for the control group, healthy epithelial tissue was a minimum of 5 cm away from the border of the tissue of ESCC. The criteria for exclusion included the following: adenocarcinoma of the esophagus, those who had undergone radiation or chemotherapy before the operation, malignant metastases to the esophagus, and individuals from other ethnic groups such as Uygur and Mongolian. Each individual’s data comprised their age, ethnicity, gender, tumor location and size, differentiation level, American Joint Committee on Cancer (AJCC) staging (based on the eighth version of ESCC provided by the AJCC and Union for International Cancer Control [UICC] in 2017), status of lymph node, vascular and nerve invasion, and the advancement of the disease. The Ethics Committee of the First Affiliated Hospital of Xinjiang Medical University granted authorization for this investigation. Prior to gathering specimens, everybody who participated provided informed consent. The follow‐up period was extended until April 2023, including the investigation of medical records and phone calls. As shown in Table S1, the clinicopathological variables considered in the present investigation included age (less than 60 years old or older), gender (male vs. female), ethnicity (Han vs. Kazakh), tumor location (upper, middle, or lower), tumor size (less than 3 vs. 3 cm or larger), differentiation (poor, moderate, or well), metastasis of lymph nodes (no vs. yes), depth of invasion (mucosa, muscularis, or full thickness), AJCC stage (I + II or III + IV), vascular invasion (no vs. yes), nerve invasion (no vs. yes), hematogenous metastasis (no vs. yes), postoperative treatment (no vs. yes).

The expression data of 82 ESCC tissues and 11 paracancerous tissues were collected from the TCGA database (https://portal.gdc.cancer.gov/).

### 2.2. Antibodies and Reagents

The primary antibodies: UBE2S (Proteintech Group, Number 14115‐1‐AP, 1:360), HIF‐1α (BIOSS, bs‐0737R, 1:400), and FOXM1 (Affinity, DF6962, 1:130). Additional reagents used in the investigation include an endogenous peroxidase blocker, a working solution of normal goat serum for sealing, 3,3‐diaminobenzidine (DAB), a working solution of horseradish enzyme‐labeled streptomycin, and a biotin‐labeled goat anti‐rabbit/anti‐mouse IgG. These reagents were acquired from Zhongshan Jinqiao Company, Beijing, China.

### 2.3. Immunohistochemistry (IHC)

The tissue chips were exposed to a temperature of 70°C in an oven for a duration of 30 min to facilitate the softening of the wax coating that covers them. Following that, the tissues were treated with xylene to remove the paraffin and then soaked in a series of ethanol solutions with concentrations of 100%, 95%, 80%, and 70% to restore their hydration. The tissues were immersed in hydrogen peroxide for a duration of 10 min. Immerse the tissue chip in a heated ethylenediaminetetraacetic acid (EDTA) repair solution (pH = 9.0) and maintain boiling for 30 min to facilitate antigen retrieval. Rinse three times with phosphate‐buffered solution (PBS) for a duration of 2 min every time. Following the application of goat serum for blocking, place the tissue chip for 20 min in a temperature‐controlled oven set at a constant 37°C. The segments underwent 90 min of incubation with primary antibodies UBE2S, HIF‐1α, and FOXM1 at 37°C. Rinse three times with PBS for a duration of 2 minutes each. Put the secondary antibody (goat anti‐rabbit/anti‐mouse IgG) in small drops and allow it to incubate in a 37°C oven for a duration of 30 min. Washing three times with PBS to thoroughly wash off unbound secondary antibodies. Finally, adding the DAB staining solution and rinse with clean water until a brownish‐yellow color on the section was observed. It was stained in hematoxylin solution for 30 s, immersed in 1% hydrochloric acid ethanol for differentiation, put into a blue‐returning solution for about 2 s, and then, stained with gradient ethanol (70%, 80%, 95%, and 100%, concentration from low to high). The segments were dehydrated in a fume chamber, then, immersed in a neutral resin gel, and mounted on glass slides.

The guidelines for interpreting IHC findings were as follows: The tissue microscope was subjected to microscopic inspection at consistent incident light intensity and compensation intensity at each spot. Five randomly chosen high‐power fields (×400) were selected. The location of staining (nucleus and/or cytoplasm) was assessed by examining the staining intensity and the percentage of positive cells. UBE2S was located in the cytoplasm and nucleus, while HIF‐1α and FOXM1 were mainly located in the nucleus. The instruction for the intensity score of staining was 0 (unstained), 1 (lightly stained, light yellow), 2 (moderately stained, brown), and 3 (heavily stained, dark brown). The rule of stained positive cells scores: 0 (<10% positive tumor cells), 1 (10%–25% positive tumor cells), 2 (26%–50% positive tumor cells), 3 (51%–75% positive tumor cells), and 4 (76%–100% positive tumor cells). The staining index was determined utilizing this formula: Multiply the percentage of positive cells by the staining intensity score. The staining indexes that were obtained include the values 0, 1, 2, 3, 4, 6, 9, and 12. The sum of the products from five randomly selected fields resulted in a total score. Subsequently, the cumulative score was divided by 5 to determine the score of IHC. High expression was regarded as an IHC score of 4 points or more, while low level of expression was described as a score below 4 points. The IHC score of the tissue chip was assessed by two pathologists in a blind manner, without any access to the participants’ medical information. All discrepancies in scoring were addressed via discussion.

### 2.4. Statistical Analysis

The relationship between medical and pathological characteristics were assessed employing the *χ* ^2^ test and Fisher’s exact test. The Spearman correlation analysis was utilized to test the connection between UBE2S, HIF‐1α, and FOXM1. Survival duration was determined by assessing progression‐free survival (PFS) and overall survival (OS). PFS refers to the duration between the first diagnosis of cancer of the esophagus in a patient and either the advancement of the tumor or the patient’s death. The investigation employed both univariate and multivariate analyses to conduct survival analysis. The Kaplan–Meier (K–M) technique and Cox hazard regression analysis were employed for this purpose. The K–M method was employed to examine the clinicopathological characteristics linked to the ESCC prognosis. The Cox hazard regression analysis employed the K–M approach to examine the independent variables linked to ESCC prognosis. The covariates of Cox hazard regression analysis consisted of UBE2S, HIF‐1α, FOXM1, gender, age, ethnicity, location and size of the tumor, level of differentiation, AJCC stage, the status of lymph node, vascular and nerve invasion, therapy postsurgery, et cetera. The study used the forward likelihood ratio (LR) approach. The findings of the Cox hazard regression analysis were presented, including the hazard ratios (HRs) and 95% CI. The Kruskal–Wallis test was employed to examine the discrepancies in expression between intraepithelial neoplasia and cancer tissues, and the outcomes were adjusted employing the Bonferroni. A probability value below 0.05 was deemed statistically significant. The data was examined and processed utilizing the SPSS statistical 25.0 program developed by IBM Corporation.

## 3. Result

### 3.1. Clinicopathologic Characteristics

The demographic information and pathological features of the 173 individuals diagnosed with ESCC who were included in the investigation are summarized in Table S1. Among the 173 patients, the majority of the patients were 60 years old or older (114/65.9%), the majority of patients were male (120/69.4%), nearly half were of Kazakh (83/48.0%), 103 patients with the middle segment (59.5%), and the majority were classified as AJCC stage I + II (127/73.4%). Out of the total of 173 patients, 78 (45.1%) individuals had comprehensive surgical procedures followed by postoperative therapy. Subjects were monitored for an average of 30 months (ranging from 1 to 128 months) and 130 (75.1%) individuals died during the monitoring time.

### 3.2. Expression of UBE2S, HIF‐1α, and FOXM1 in ESCC and Normal Esophageal Tissues

The expression of UBE2S, HIF‐1α, and FOXM1 in the TCGA database were higher in ESCC samples (*p* < 0.001; Figure 1). Employing IHC, we detected UBE2S, HIF‐1α, and FoxM1 in both the neighboring healthy esophageal mucosa epithelium and ESCC tissues. Figure 2 displays the typical IHC photos of UBE2S, HIF‐1α, and FOXM1. The outcomes of the IHC staining exhibited that the pattern of UBE2S expression was evident, namely, in the cytoplasm and nucleus, HIF‐1α and FOXM1 expression were major situated in the cancer cells nucleus (Figure 2A–I). HIF‐1α, UBE2S, and FOXM1 were either absent or only present in the basal layer cells in normal tissues of the esophagus. In the ESCC, the UBE2S expression rate was 72.3% (125/173), whereas in the normal mucosa of the esophagus, the rate of expression was 32.4% (56/173). This variation was found to be statistically significant (Table 1). Among the 173 cases of ESCC, 128 cases had a high expression for HIF‐1α expression, and 45 individuals had a low expression, HIF‐1α high expression rate in ESCC of 74.0%. Among the 173 cases of adjacent normal esophageal mucosa epithelium, 31 cases had a high expression for HIF‐1α expression, HIF‐1α expression rate in adjacent normal esophageal mucosa epithelium of 17.9%, and statistically, the variations were significant (Table 1).

Figure 1Expression of UBE2S, HIF‐1α, and FOXM1 in ESCC in the TCGA database ( ^∗∗∗∗^ *p* < 0.001). (A) The UBE2S expression difference between 82 ESCC tissues and 11 paracancerous tissues. (B) Differences in HIF‐1α expression between ESCC tissues (*n* = 82) and normal esophageal tissues (*n* = 11). (C) The FOXM1 expression difference between 82 ESCC tissues and 11 paracancerous tissues.

**Figure:**
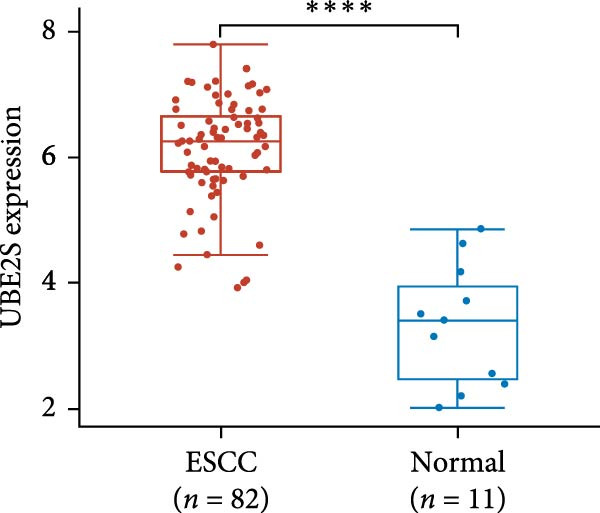
(A)

**Figure:**
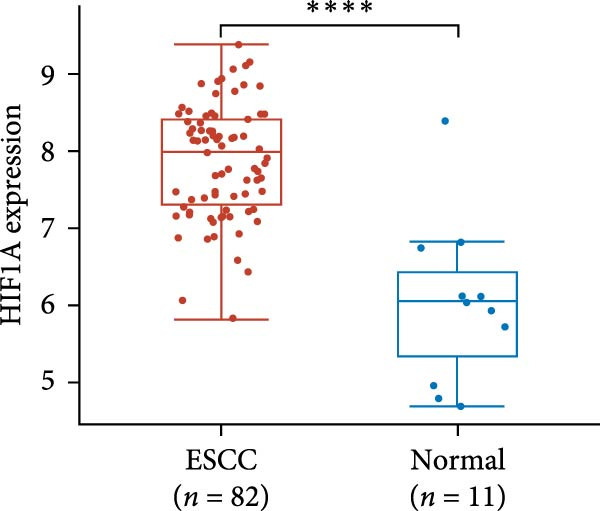
(B)

**Figure:**
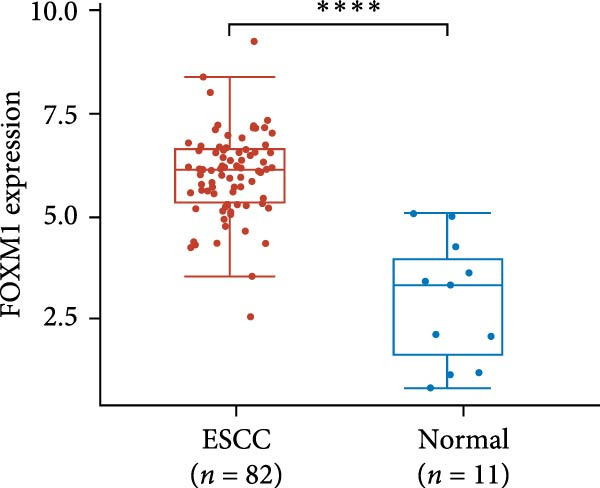
(C)

Figure 2Expression of UBE2S, HIF‐1α, and FOXM1 in ESCC and normal esophageal tissues. Immunohistochemical staining of ESCC for (A) UBE2S (high expression); (B) HIF‐1α (high expression); (C) FOXM1 (high expression); (D) UBE2S (low expression); (E) HIF‐1α (low expression); (F) FOXM1 (low expression). Staining of normal esophageal tissues for (G) UBE2S (low expression); (H) HIF‐1α (low expression); (I) FOXM1 (low expression; magnification, ×100 in magnified window; scale bar: 200 *μ*m).

**Figure:**
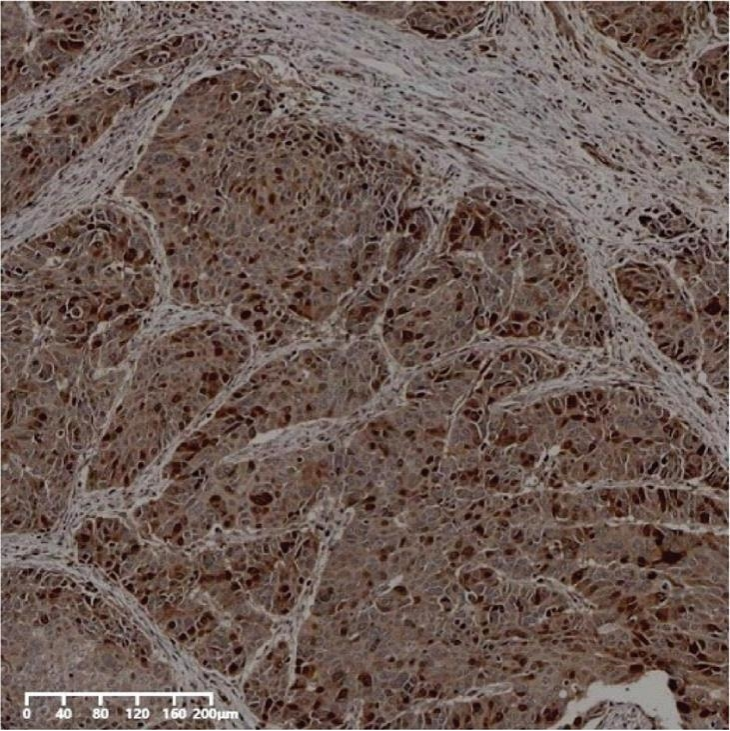
(A)

**Figure:**
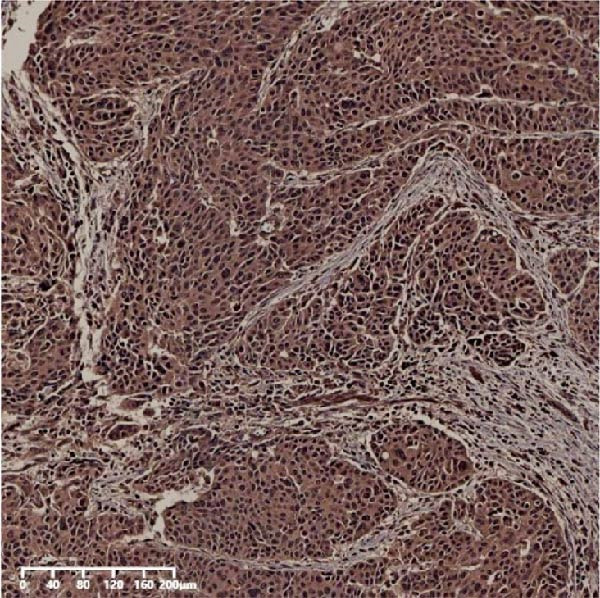
(B)

**Figure:**
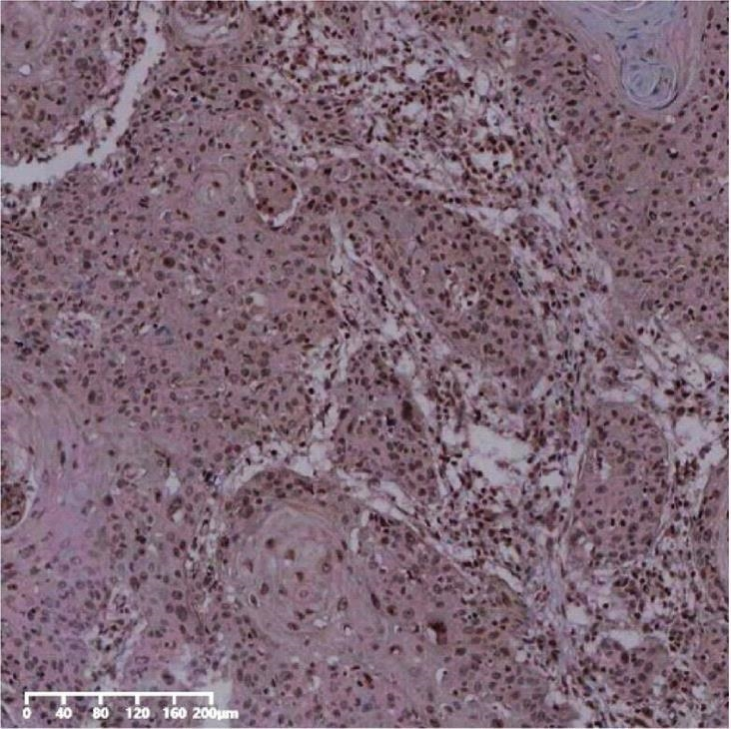
(C)

**Figure:**
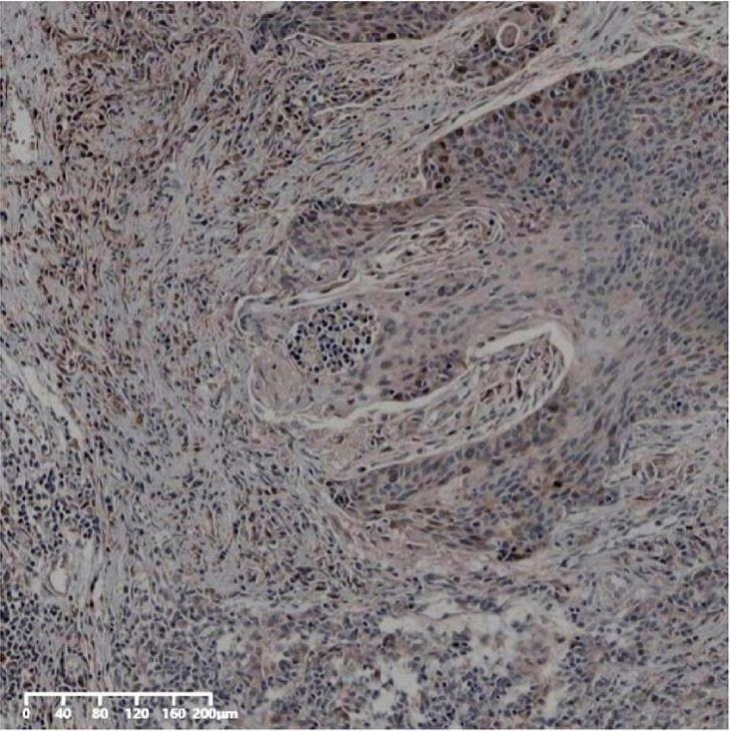
(D)

**Figure:**
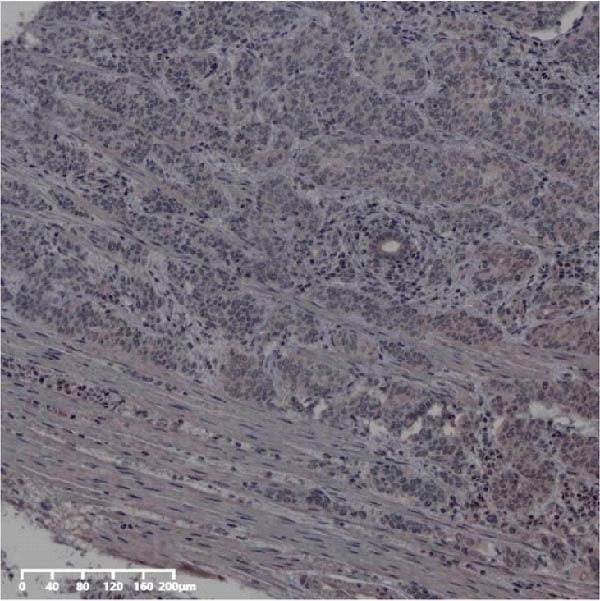
(E)

**Figure:**
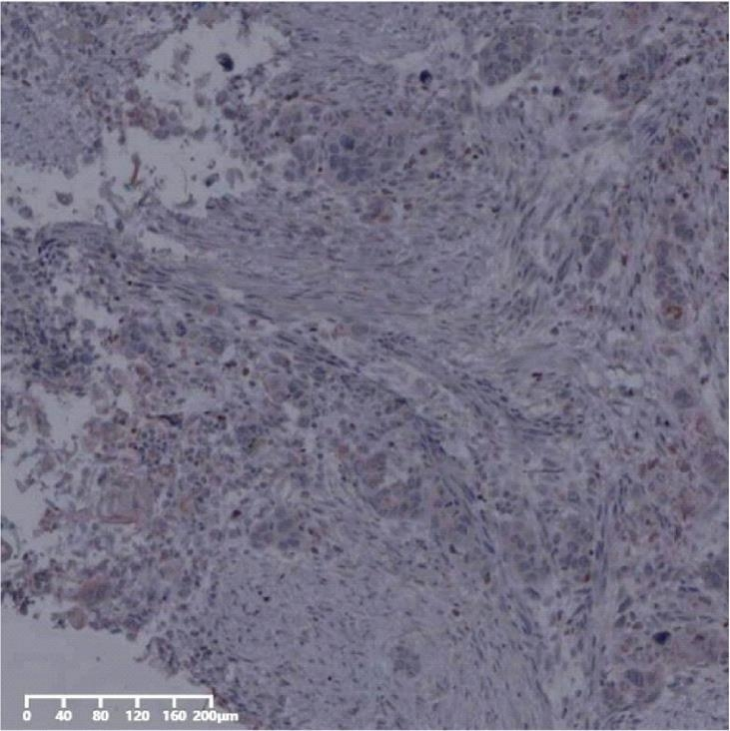
(F)

**Figure:**
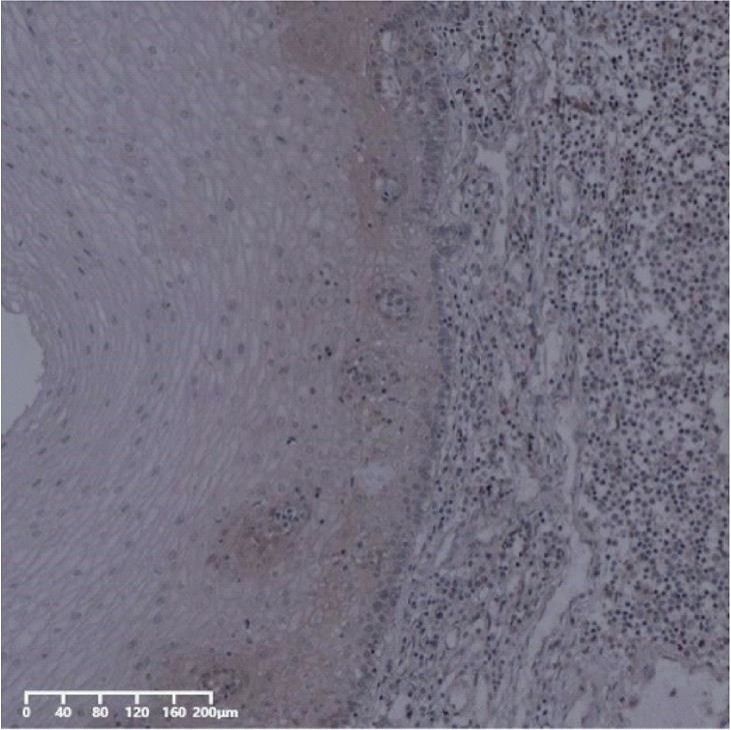
(G)

**Figure:**
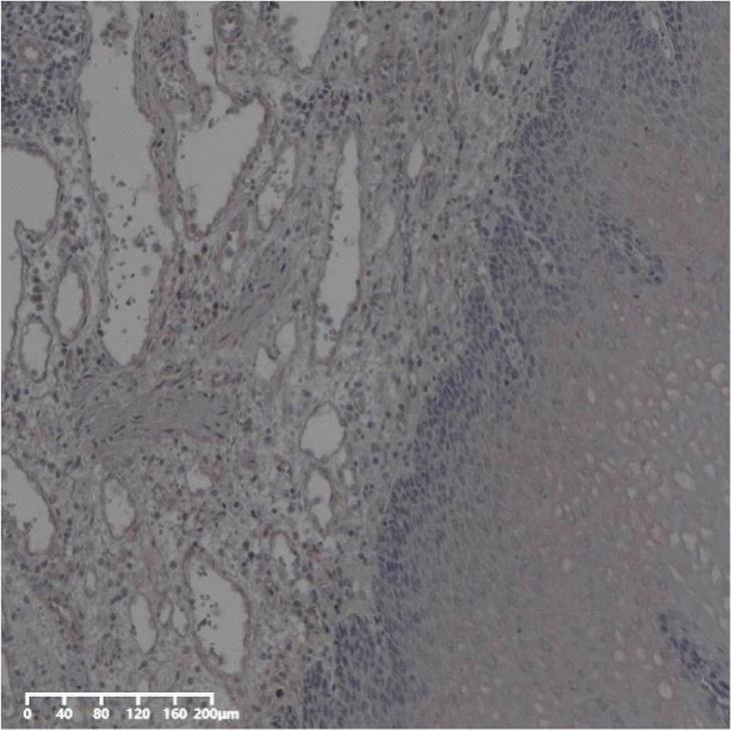
(H)

**Figure:**
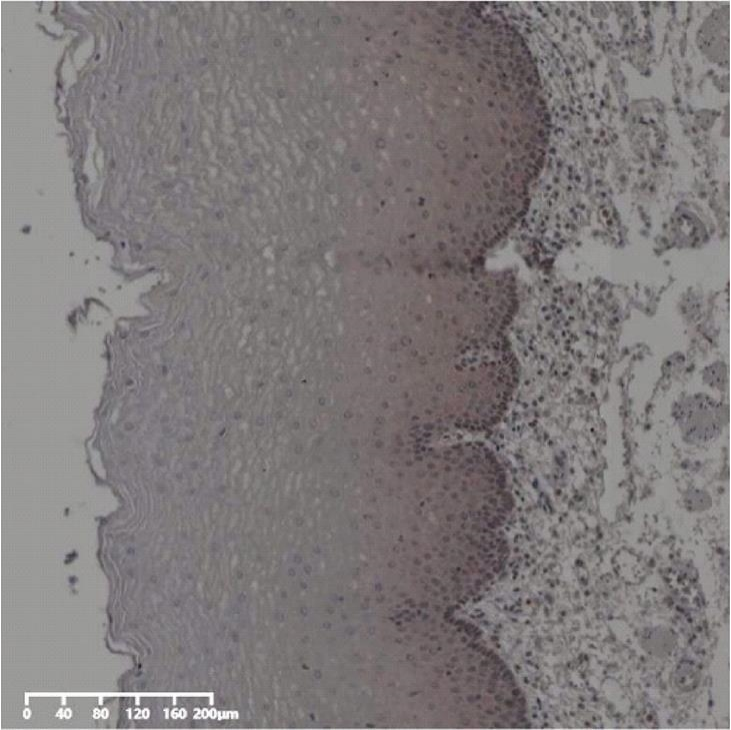
(I)

**Table 1:** UBE2S, HIF‐1α, and FOXM1 expression in ESCC and adjacent tissues.

Protein	Low expression	High expression	Sum	*p*‐Value
UBE2S				
Cancer	48	125	173	0.044
Control	117	56	173	—
Sum	165	181	346	—
HIF‐1α				
Cancer	45	128	173	0.022
Control	142	31	173	—
Sum	187	159	346	—
FOXM1				
Cancer	61	112	173	0.024
Control	165	8	173	—
Sum	226	120	346	—

In ESCC, the high expression rate of FOXM1 was 64.7% (112/173), while in the neighboring normal tissues, FOXM1’s elevated expression rate was only 4.6% (8/173), and statistically, variations were significant (Table 1). UBE2S, HIF‐1α, and FOXM1 staining and scoring in ESCC were stronger, whereas those in normal tissues of the esophagus were weaker. All three were upregulated proteins in cancer tissue.

### 3.3. Analysis of Correlation Between UBE2S, HIF‐1α, and FOXM1 Proteins Expression and Clinicopathological Features in ESCC

The relationship between increased levels of the three proteins and the clinicopathological characteristics was evaluated in the 173 subjects with ESCC. The correlation between expression data and clinicopathological features was detected. The investigation found a significant relationship between elevated levels of UBE2S and ethnicity (*p* < 0.001) in addition to the tumor site (*p* = 0.021; Table 2). Additionally, excessive HIF‐1α expression was shown to be correlated with metastasis from lymph nodes (*p* = 0.017), while increased FOXM1 expression was also correlated with metastasis of lymph nodes (*p* = 0.015).

**Table 2:** UBE2S, HIF‐1α, and FOXM1 in esophageal squamous cell carcinoma and their relationship with clinicopathological features.

Clinical parameters	UBE2S [number (%)]		*p*‐Value	HIF‐1α [number (%)]		*p*‐Value	FOXM1 [number (%)]		*p*‐Value
	Low expression	High expression		Low expression	High expression		Low expression	High expression	
Age									
<60	11 (18.6)	48 (81.4)	0.054	18 (26.9)	49 (73.1)	0.839	24 (35.8)	43 (64.2)	0.902
≥60	37 (32.5)	77 (67.5)	—	27 (25.5)	79 (74.5)	—	37 (34.9)	69 (65.1)	—
Gender									
Male	36 (30.0)	84(70.0)	0.319	31 (25.8)	89 (74.2)	0.936	41 (34.2)	79 (65.8)	0.651
Female	12 (22.6)	41(77.4)	—	14 (26.4)	39 (73.6)	—	20 (37.7)	33 (62.3)	—
Ethnicity									
Han	36 (40.0)	54 (60.0)	<0.001	20 (22.2)	70 (77.8)	0.237	30(33.3)	60 (66.7)	0.581
Kazakh	12 (14.5)	71 (85.5)	—	25 (30.1)	58 (69.9)	—	31 (37.3)	52 (62.7)	—
Tumor location									
Upper	6 (66.7)	3 (33.3)	0.021	3 (33.3)	6 (66.7)	0.703	4 (44.4)	5 (55.6)	0.638
Middle	24 (23.3)	79 (76.7)	—	28 (27.2)	75 (72.8)	—	34 (33.0)	69 (67.0)	—
Lower	18 (29.5)	43 (70.5)	—	14 (23.0)	47 (77.0)	—	23 (37.7)	38 (62.3)	—
Tumor size(cm)									
<3	18 (32.7)	37 (67.3)	0.318	16 (29.1)	39 (70.9)	0.528	23 (41.8)	32 (58.2)	0.218
≥3	30 (25.4)	88 (74.6)	—	29 (24.6)	89 (75.4)	—	38 (32.2)	80 (67.8)	—
Differentiation									
Poor	10 (37.0)	17 (63.0)	0.446	5 (18.5)	22 (81.5)	0.052	12 (44.4)	15 (55.6)	0.357
Moderate	24 (24.7)	73 (75.3)	—	21 (21.6)	76 (78.4)	—	30 (30.9)	67 (69.1)	—
Well	14 (28.6)	35 (71.4)	—	19 (38.8)	30 (61.2)	—	19 (38.8)	30 (61.2)	—
Lymph metastasis									
No	33 (27.0)	89 (73.0)	0.752	38 (31.1)	84 (68.9)	0.017	50 (41.0)	72 (59.0)	0.015
Yes	15 (29.4)	36 (70.6)	—	7 (13.7)	44 (86.3)	—	11 (21.6)	40 (78.4)	—
Invasive depth									
Mucosa	1 (20.0)	4 (80.0)	0.948	1 (20.0)	4 (80.0)	0.294	2 (40.0)	3 (60.0)	0.316
Muscularis	19 (27.1)	51 (72.9)	—	14 (20.0)	56 (80.0)	—	20 (28.6)	50 (71.4)	—
Full thickness	28 (28.6)	70 (71.4)	—	30 (30.6)	68 (69.4)	—	39(39.8)	59 (60.2)	—
AJCC stage									
I + II	32 (25.2)	95 (74.8)	0.213	37 (29.1)	90 (70.9)	0.120	49 (38.6)	78 (61.4)	0.129
III + IV	16(34.8)	30(65.2)	—	8 (17.4)	38 (82.6)	—	12 (26.1)	34 (73.9)	—
Vascular invasion									
No	42 (29.4)	101 (70.6)	0.297	37 (25.9)	106 (74.1)	0.928	55 (38.5)	88 (61.5)	0.054
Yes	6 (20.0)	24 (80.0)	—	8 (26.7)	22 (73.3)	—	6 (20.0)	24 (80.0)	—
Nerve invasion									
No	38 (26.6)	105 (73.4)	0.452	39 (27.3)	104 (72.7)	0.409	51 (35.7)	92 (64.3)	0.808
Yes	10 (33.3)	20 (66.7)	—	6 (20.0)	24 (80.0)	—	10 (33.3)	20 (66.7)	—
Hematogenous metastasis									
No	39 (26.2)	110 (73.8)	0.250	39 (26.2)	110 (73.8)	0.903	51 (34.2)	98 (65.8)	0.479
Yes	9 (37.5)	15 (62.5)	—	6 (25.0)	18 (75.0)	—	10 (41.7)	14 (58.3)	—
Postoperative treatment									
No	22 (23.2)	73 (76.8)	0.137	21 (22.1)	74 (77.9)	0.196	30 (31.6)	65 (68.4)	0.263
Yes	26 (33.3)	52 (66.7)	—	24 (30.8)	54 (69.2)	—	31 (39.7)	47 (60.3)	—

*Note:* The relationships between medical and pathological characteristics were assessed employing the *χ* ^2^ test and Fisher’s exact test.

___*p* < 0.05 was considered statistically significant.

### 3.4. Connection of UBE2S, HIF‐1α, and FOXM1 Protein Expression

**Figure 3:**
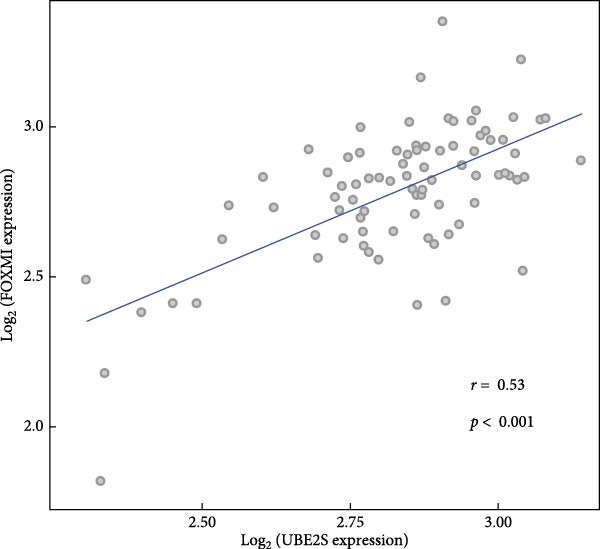
The relationship between UBE2S expression and FOXM1 expression in the TCGA database was analyzed by Spearman in ESCC. In the TCGA database the UBE2S expression was positively correlated with FOXM1 (*r* = 0.53; *p* < 0.001) in ESCC samples.

The UBE2S expression was positively correlated with FOXM1 (*r* = 0.53; *p* < 0.001) in the TCGA database in ESCC samples (Figure 3). A Spearman correlational investigation was conducted on a sample of 173 subjects of ESCC. A significant positive connection was detected between UBE2S and HIF‐1α expression (*r* = 0.192; *p* = 0.012). UBE2S expression was found to have a positive relationship with FOXM1, *r* of 0.245, and a *p*‐value of 0.001. Tables 3 and 4 demonstrate a significant positive relationship (*r* = 0.307; *p* < 0.001) between HIF‐1α and FOXM1 expression.

**Table 3:** Correlation between UBE2S, HIF‐1α, and FOXM1 in ESCC.

Protein	UBE2S expression	
	*r*	*p*‐Value
HIF‐1α expression	0.192	0.012
FOXM1 expression	0.245	0.001

*Note:* The Spearman correlation analysis was utilized to test the connection between UBE2S, HIF‐1α, and FOXM1 in ESCC.

**Table 4:** Correlation between HIF‐1α and FOXM1 in ESCC.

Protein	HIF‐1α expression	
	*r*	*p*‐Value
FOXM1 expression	0.307	<0.001

*Note:* The Spearman correlation analysis was utilized to test the connection between HIF‐1α and FOXM1 in ESCC.

### 3.5. Predictive Factors for OS and PFS

The connection between protein level of expression and the survival rate for those with ESCC was examined utilizing the K–M technique. The K–M univariate analysis demonstrated significant correlations between the OS rate of ESCC subjects and many factors, including the level of differentiation (*p* = 0.027; Figure 4A), metastases of lymph nodes (*p* < 0.001; Figure 4B), AJCC stage (*p* = 0.017; Figure 4C), and postoperatively therapy (*p* < 0.001; Figure 4D). Nevertheless, there was not a link between it and gender, age, ethnicity, site and size of the tumor, depth of invasion, vascular and nerve invasion, and hematogenous metastasis. Gender (*p* = 0.037; Figure 4E), degree of differentiation (*p* = 0.018; Figure 4F), metastasis of lymph nodes (*p* < 0.001; Figure 4G), AJCC stage (*p* = 0.003; Figure 4H), and postoperatively therapy (*p* = 0.001; Figure 4I) were determined to have a significant association with PFS. Subjects who had elevated expressions of UBE2S (*p* = 0.002; Figure 4J), HIF‐1α (*p* = 0.002; Figure 4K), and FOXM1 (*p* < 0.001; Figure 4L) had a lower OS. Additionally, those with increased UBE2S (*p* = 0.002; Figure 4M), HIF‐1α (*p* = 0.007; Figure 4N), and FOXM1 (*p* < 0.001; Figure 4O) expressions had poorer PFS (Figure 4).

Figure 4Kaplan–Meier survival analysis for overall survival and progression‐free survival. (A) In ESCC, patients with low differentiation (*p* = 0.027) had shorter OS. (B) Individuals with metastases of lymph node had a shorter of OS (*p* < 0.001). (C) Subjects with AJCC Stages I and II had better OS than AJCC Stages III and IV in ESCC (*p* = 0.017). (D) The OS of individuals with ESCC underwent radiotherapy and chemotherapy following operation was longer (*p* < 0.001). (E) PFS of male patients was shorter (*p* = 0.037). (F) Individuals with low differentiation (*p* = 0.018) had shorter PFS. (G) Individuals with lymph node metastases had a shorter of PFS (*p* < 0.001). (H) Subjects with AJCC Stages I and II had better PFS than AJCC Stages III and IV in ESCC (*p* = 0.003). (I) The PFS of subjects with ESCC who underwent radiotherapy and chemotherapy following operation was longer (*p* = 0.001). (J) The increased UBE2S expression had a poor OS in ESCC (*p* = 0.002). (K) The elevated HIF‐1α expression had a poor OS in ESCC (*p* = 0.002). (L) The high expression of FOXM1 had a poor OS in ESCC (*p* < 0.001). (M) The elevated UBE2S expression had a poor PFS in ESCC (*p* = 0.002). (N) The increased HIF‐1α expression had a poor PFS in ESCC (*p* = 0.007). (O) The increased FOXM1 expression had a poor PFS in ESCC (*p* < 0.001). ESCC, esophageal squamous cell carcinoma; OS, overall survival; PFS, progression‐free survival.

**Figure:**
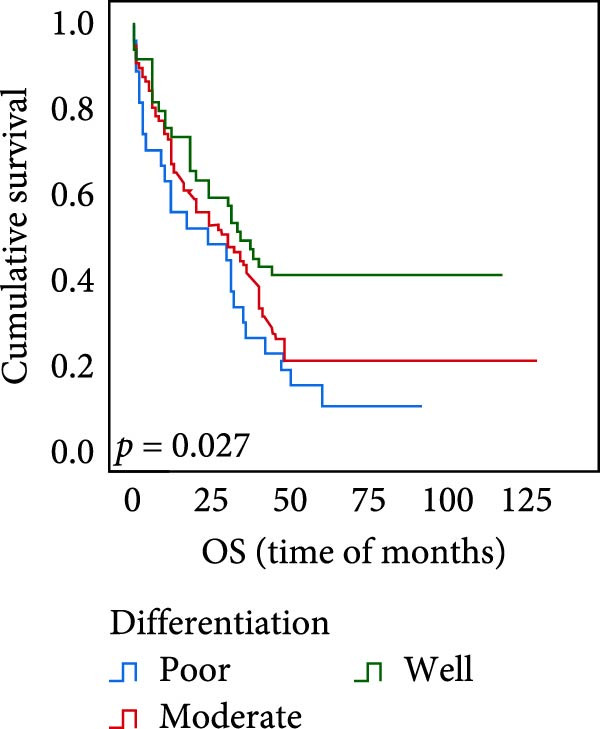
(A)

**Figure:**
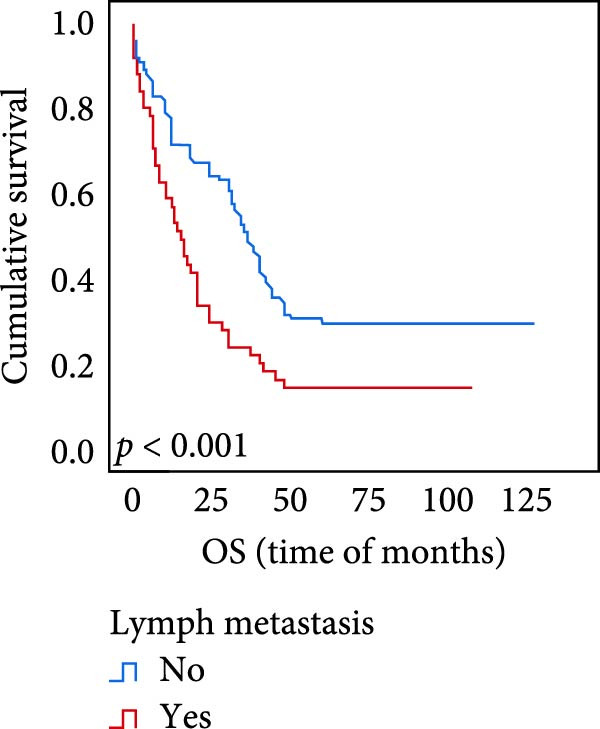
(B)

**Figure:**
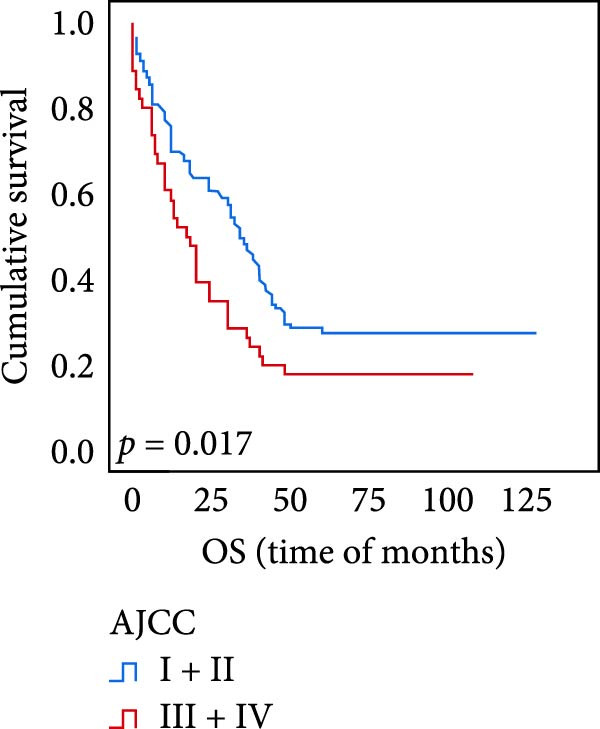
(C)

**Figure:**
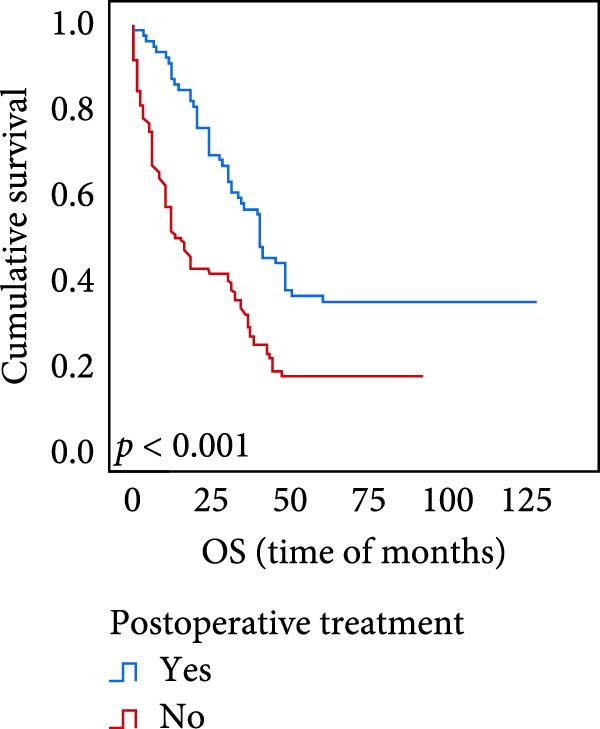
(D)

**Figure:**
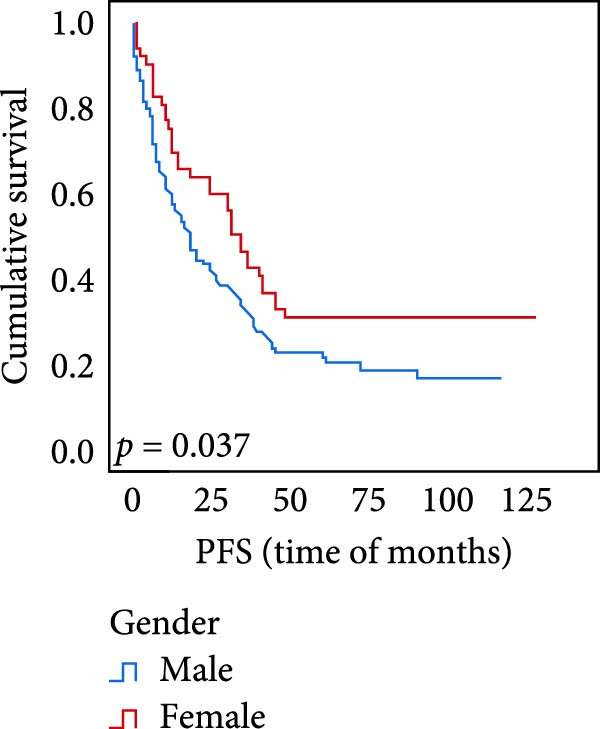
(E)

**Figure:**
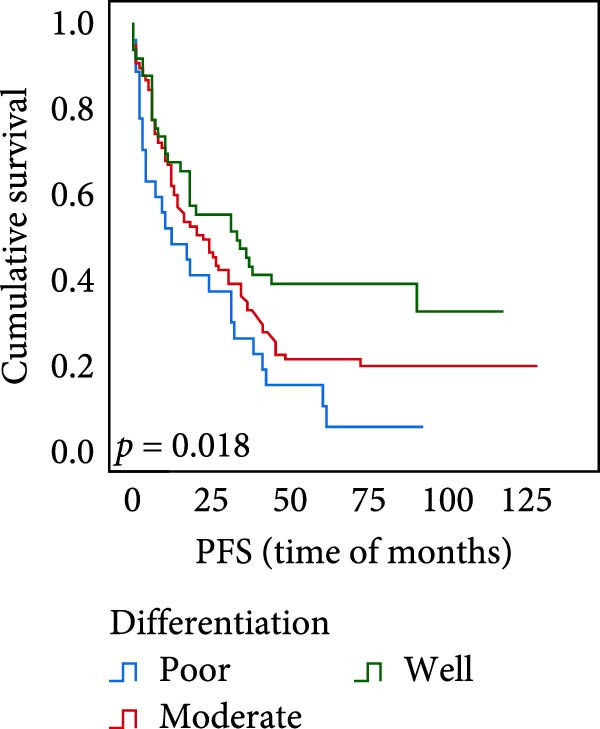
(F)

**Figure:**
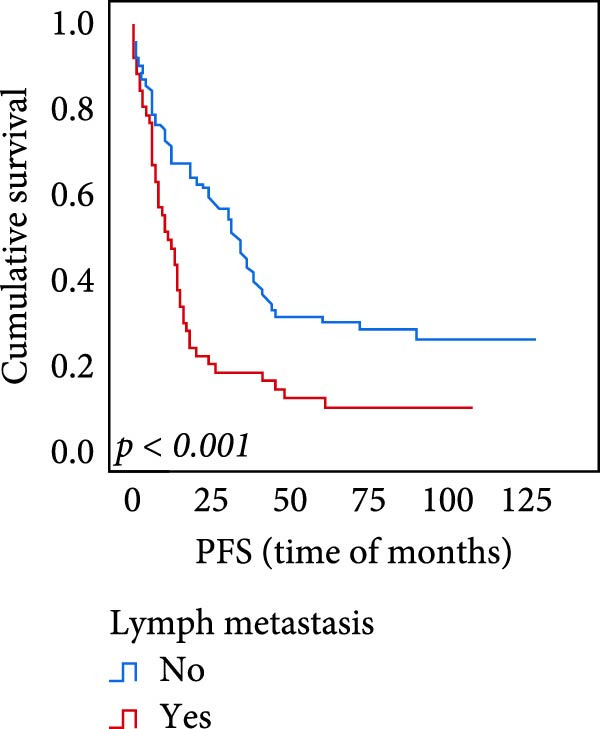
(G)

**Figure:**
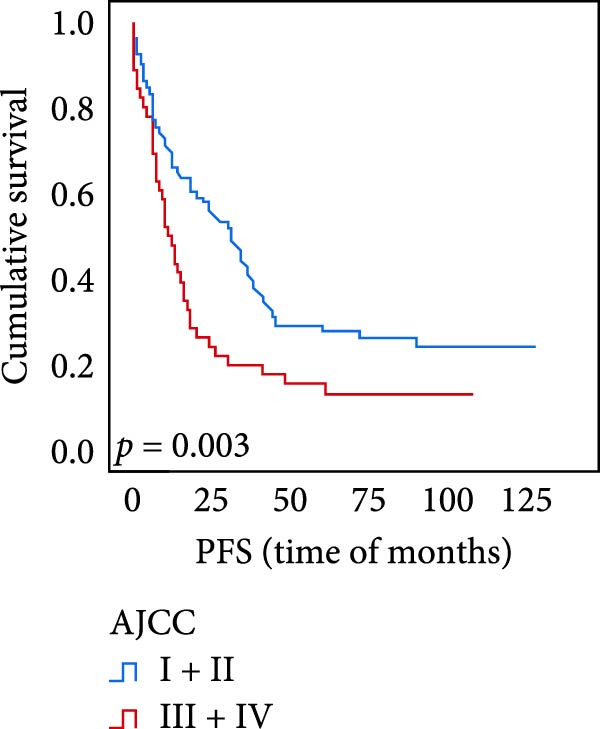
(H)

**Figure:**
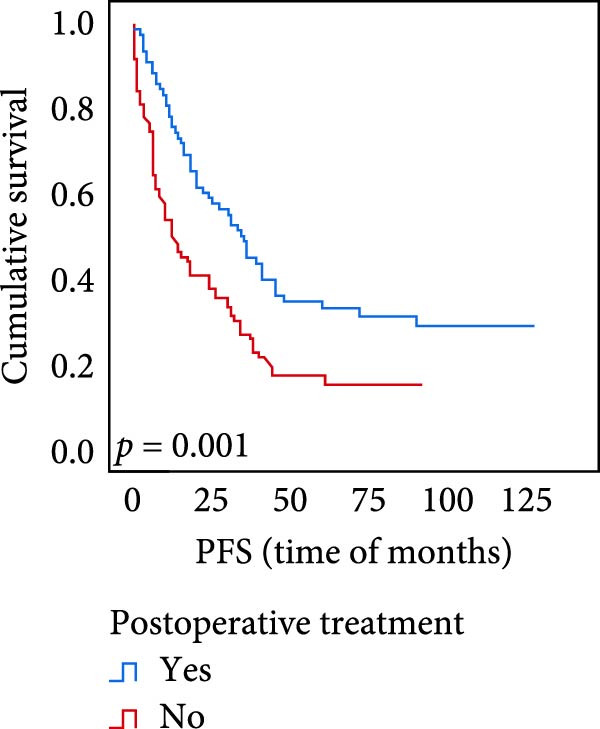
(I)

**Figure:**
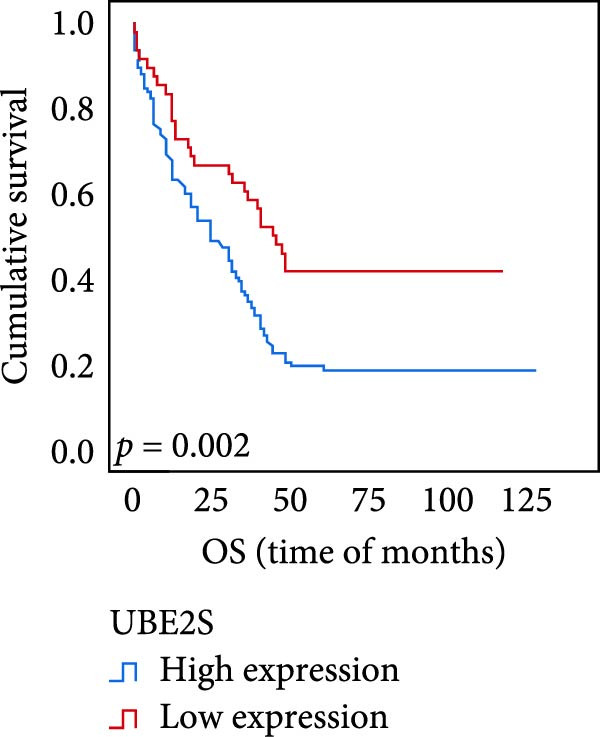
(J)

**Figure:**
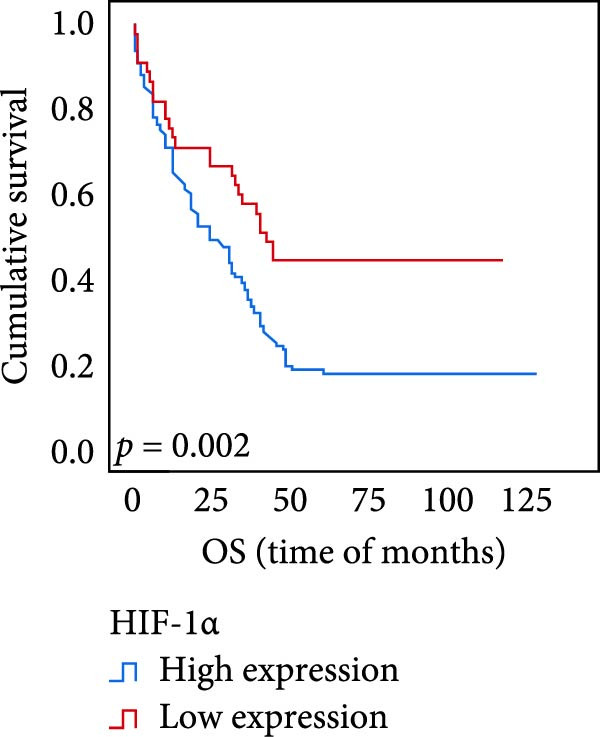
(K)

**Figure:**
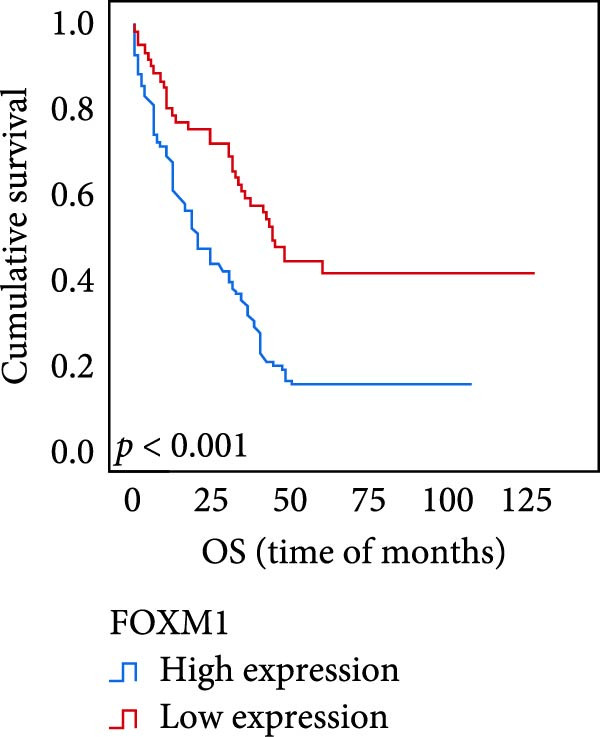
(L)

**Figure:**
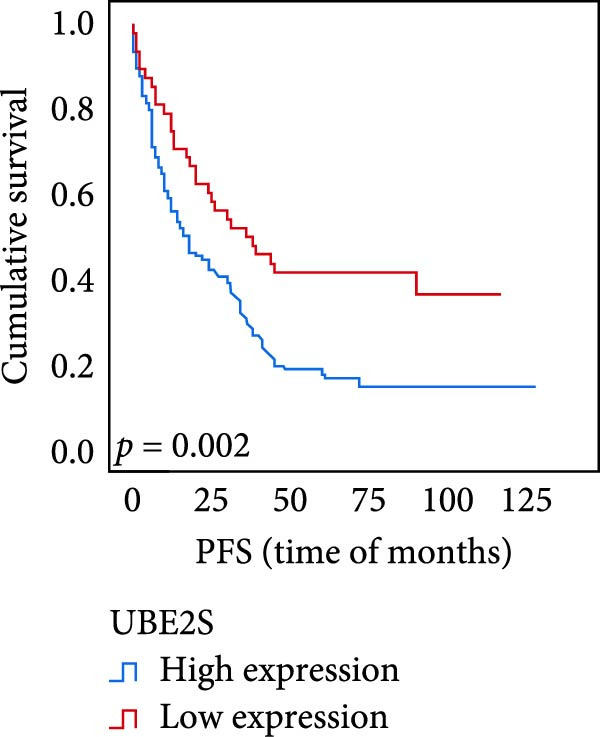
(M)

**Figure:**
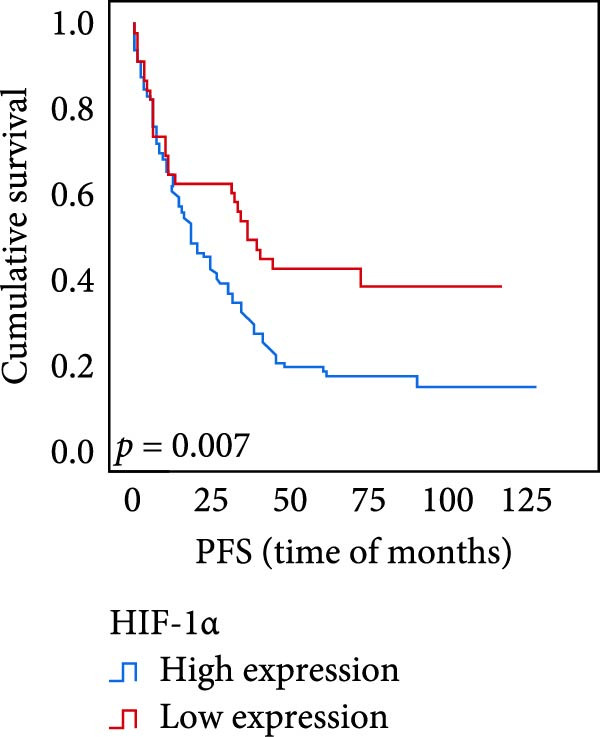
(N)

**Figure:**
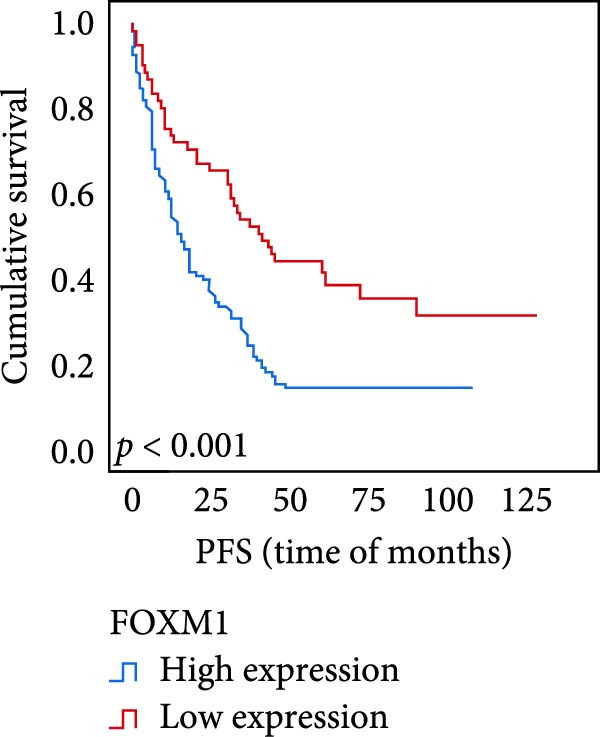
(O)

Variables with *p*  < 0.15 in the univariate analysis were used for the univariate analysis to ensure that no vital variables went undetected. In Table 5, the Cox multivariate regression analysis exhibited that lymph node metastasis (95% confidence interval [CI]: 1.359–2.909, HR: 1.988, *p* < 0.001), high UBE2S (95% CI: 1.088–2.560, HR: 1.699, *p* = 0.019), and FOXM1 (95% CI: 1.244–2.784, HR: 1.861, *p* = 0.003) expression were risk factors affecting OS of ESCC. Moreover, postoperative treatment (95% CI: 0.303–0.626, HR: 0.435, *p* < 0.001) was a defensive factor for OS of ESCC. Lymph node metastasis (95% CI: 1.458–3.095, HR: 2.124, *p* < 0.001), hematogenous metastasis (95% CI: 1.237–3.367, HR: 2.041, *p* = 0.005), high UBE2S (95% CI: 1.126–2.625, HR: 1.719, *p* = 0.012), and FOXM1 (95% CI: 1.181–2.594, HR: 1.750, *p* = 0.005) expression were also discovered to be risk factors impacting PFS. Besides, postoperative treatment (95% CI: 0.307–0.652, HR: 0.447, *p* < 0.001) was discovered to be a protective factor for PFS of ESCC.

**Table 5:** Multivariate analysis of factors linked with OS and PFS for ESCC.

Characteristics	OS		PFS	
	HR (95% CI)	*p*‐Value	HR (95% CI)	*p*‐Value
Gender (male vs. female)	–	–	–	–
Differentiation (PD vs. MD vs. WD)	–	–	–	–
Lymph metastasis (no vs. yes)	1.988 (1.359–2.909)	<0.001	2.124 (1.458–3.095)	<0.001
AJCC stage (I + II vs. III + IV)	–	–	–	–
Hematogenous metastasis (no vs. yes)	–	–	2.041 (1.237–3.367)	0.005
Postoperative treatment (no vs. yes)	0.435 (0.303–0.626)	<0.001	0.447 (0.307–0.652)	<0.001
UBE2S (low expression vs. high expression)	1.699 (1.088–2.560)	0.019	1.719 (1.126–2.625)	0.012
HIF‐1α (low expression vs. high expression)	–	–	–	–
FOXM1 (low expression vs. high expression)	1.861 (1.244–2.784)	0.003	1.750 (1.181–2.594)	0.005

*Note:* The Cox hazard regression analysis was employed for multivariate analyses to conduct survival analysis.

### 3.6. The Expression of UBE2S, HIF‐1α, and FOXM1 Affected the OS Rate and PFS Time of Postoperative Chemotherapy Patients

Out of the total number of those participating in our investigation, 78 individuals, accounting for 45.1% of the sample, were administered chemotherapy after their surgery. Out of the total cases, UBE2S demonstrated elevated expression in 52 subjects (66.7%) and was negative in 26 cases. The prevalence of HIF‐1α was 69.2%, with 54 cases testing positive and 24 individuals testing negative. FOXM1 expression was elevated in 47 subjects (60.3%) and absent in 31 cases.

The results of univariate analysis, as shown in Figure 5, the time of OS (*p* < 0.001; Figure 5A) and PFS (*p* < 0.001; Figure 5B) in patients with low expression of UBE2S was significantly prolonged after postoperative treatment; the time of OS (*p* < 0.001; Figure 5C) and PFS (*p* < 0.001; Figure 5D) in patients with low expression of HIF‐1α was significantly prolonged after postoperative treatment; the time of OS (*p* < 0.001; Figure 5E) and PFS (*p* < 0.001; Figure 5F) in patients with low expression of FOXM1 was significantly prolonged after postoperative treatment.

Figure 5OS and PFS of individuals with ESCC following chemotherapy were analyzed by the Kaplan–Meier technique. (A) Prolonged overall survival in individuals with UBE2S low expression combined with chemoradiotherapy. (B) Prolonged progression‐free survival in patients with UBE2S low expression combined with chemoradiotherapy. (C) Prolonged overall survival in individuals with HIF‐1α low expression combined with chemoradiotherapy. (D) Prolonged progression‐free survival in subjects with HIF‐1α low expression combined with chemoradiotherapy. (E) Prolonged overall survival in patients with FOXM1 low expression combined with chemoradiotherapy. (F) Prolonged progression‐free survival in individuals with FOXM1 low expression combined with chemoradiotherapy.

**Figure:**
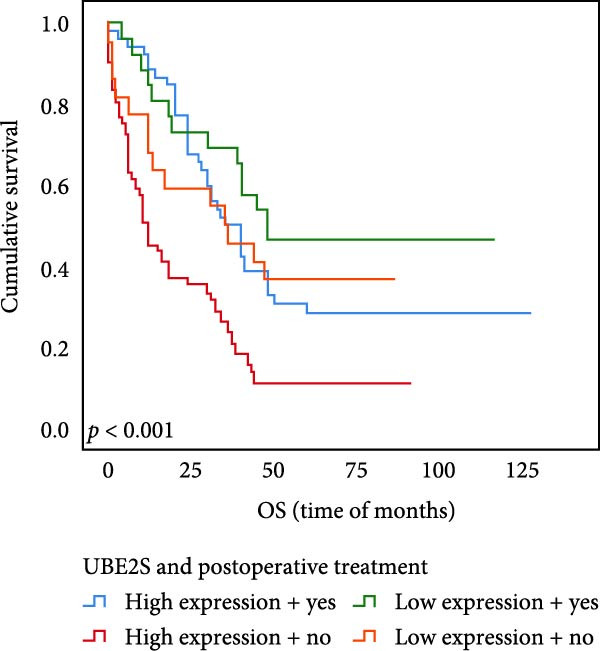
(A)

**Figure:**
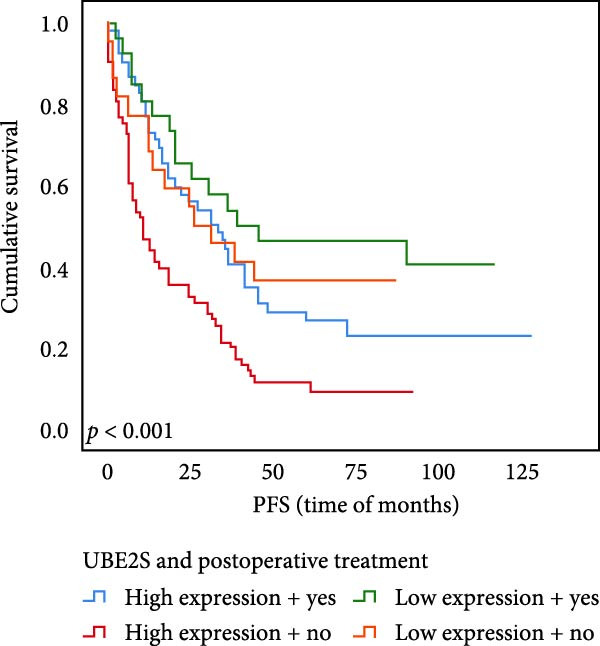
(B)

**Figure:**
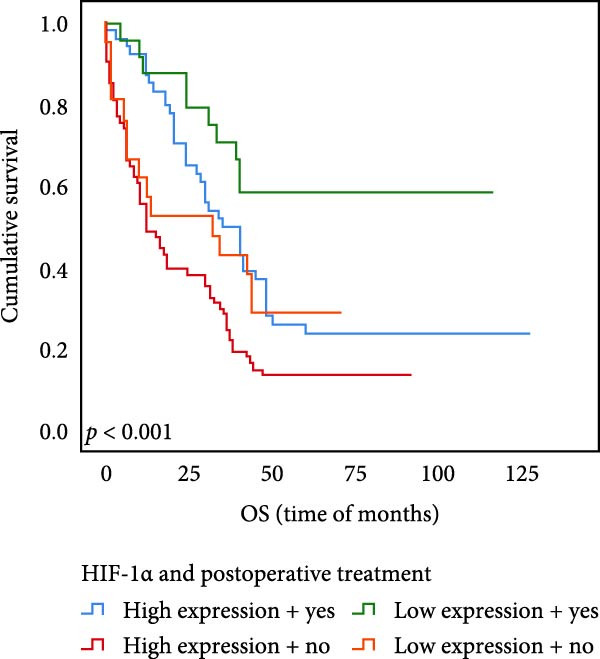
(C)

**Figure:**
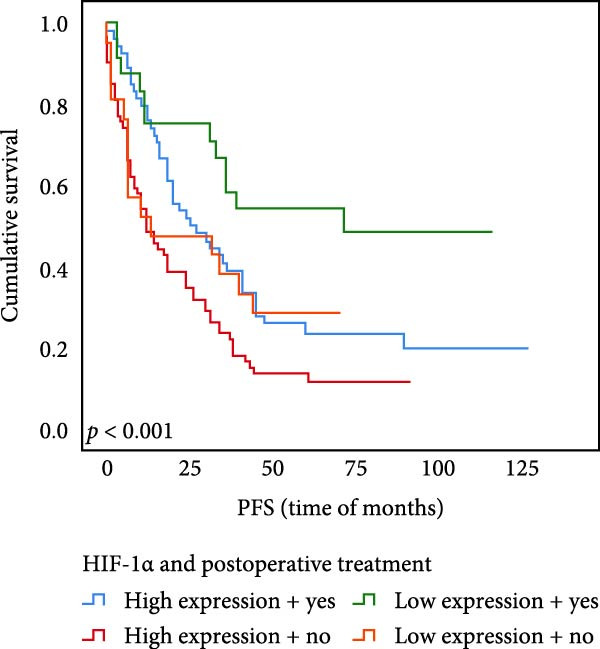
(D)

**Figure:**
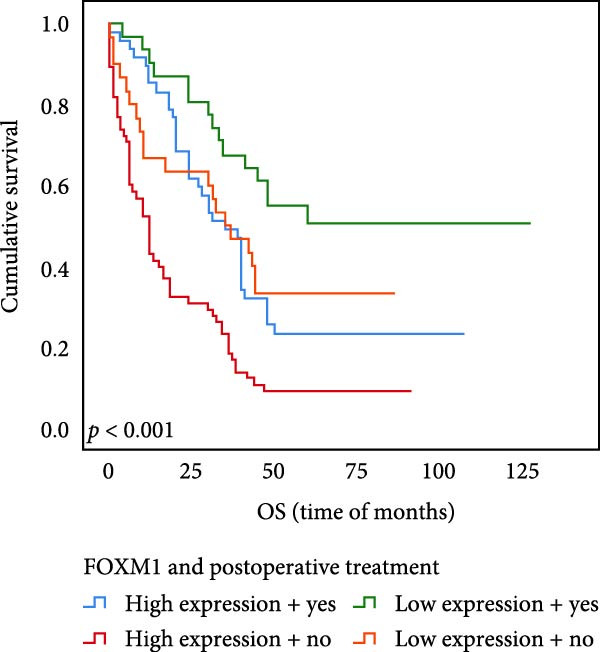
(E)

**Figure:**
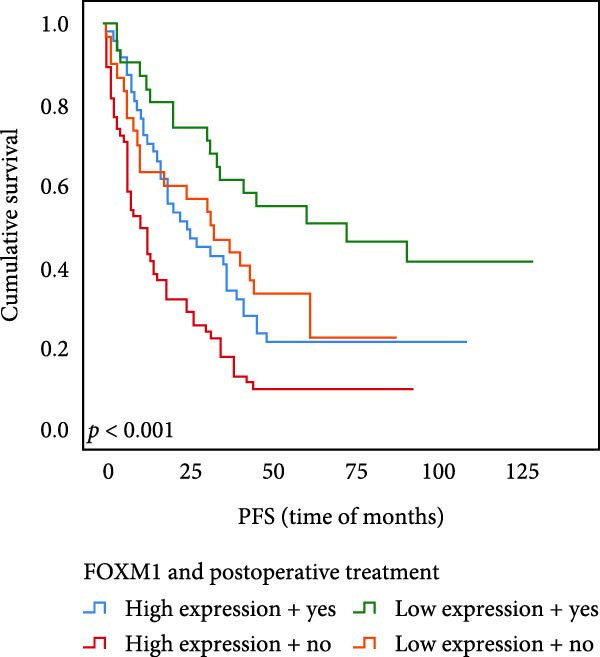
(F)

### 3.7. UBE2S, HIF‐1α, and FOXM1 Coexpression Impacts on Prognosis of Individuals With ESCC

Univariate survival analysis showed that compared with other groups, the OS and PFS (*p* < 0.001 for Figure 6A,B) of subjects coexpressed with UBE2S, HIF‐1α, and FOXM1 in ESCC were significantly shortened.

Figure 6OS and PFS analysis of individuals UBE2S, HIF‐1α, and FOXM1 co‐expression with ESCC utilizing the Kaplan–Meier technique. (A) Individuals with elevated UBE2S/HIF‐1α/FOXM1 expression had shortened overall survival. (B) Subjects with high UBE2S/HIF‐1α/FOXM1 expression had shortened progression‐free survival.

**Figure:**
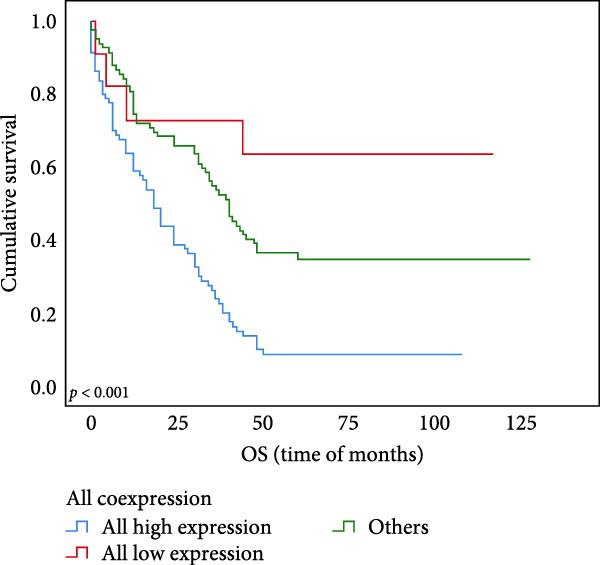
(A)

**Figure:**
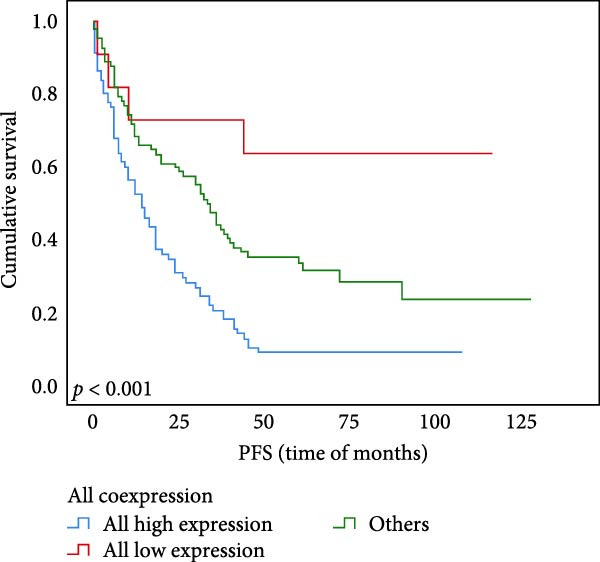
(B)

### 3.8. Differential Expression of UBE2S, HIF‐1α, and FOXM1 in Esophageal Intraepithelial Neoplasia and ESCC.

Forty‐nine cases were selected from a total of 173 cancer tissue cases as the control group for HIN and LIN using a simple random sampling method. The IHC method was carried out to observe whether there were differences in the expressions of UBE2S, HIF‐1α, and FOXM1 in low‐grade intraepithelial neoplasia (LIN), high‐grade intraepithelial neoplasia (HIN), and the normal epithelial tissues of the esophagus. The expression of UBE2S in HIN and LIN was situated in the cytoplasm and nucleus, and the expression of HIF‐1α and FOXM1 in HIN and LIN was primarily situated in the nucleus of tumor cells (Figure 7). In normal esophageal tissues, UBE2S, HIF‐1α, and FOXM1 were not expressed or only expressed in basal cells. Kruskal–Wallis test was employed to compare the protein expression differences of UBE2S, HIF‐1α, and FOXM1 in the adjacent tissues, HIN, LIN, and cancer tissues. Table 6 shows that the high UBE2S expression rates in the adjacent tissues, LIN, HIN, and ESCC were 22.4% (11/49), 44.9% (22/49), 73.5% (36/49), and 75.5% (37/49), respectively. The outcomes of pairwise comparison exhibited that the positive UBE2S rates in ESCC (*p* < 0.001) and HIN (*p* < 0.001) were greater than those in adjacent tissues. The positive rates of UBE2S in ESCC (*p* = 0.015) and HIN (*p* = 0.028) were greater than those in LIN. The high HIF‐1α protein expression rates in paraneoplastic, LIN, HIN, and ESCC were 14.3% (7/49), 30.6% (15/49), 61.2% (30/49), and 63.3% (31/49), respectively. The results of pairwise comparison exhibited that the HIF‐1α positive rates in ESCC (*p* < 0.001) and HIN (*p* < 0.001) were greater than those in adjacent tissues. The positive rates of HIF‐1α in ESCC (*p* = 0.007) and HIN (*p* = 0.013) were more than those in LIN. The high FOXM1 protein expression rates in paraneoplastic, LIN, HIN, and ESCC were 18.4% (9/49), 24.5% (12/49), 46.9% (23/49), and 71.4% (35/49), respectively. The outcomes of pairwise comparison exhibited that the positive rates of FOXM1 in ESCC (*p* < 0.001) and HIN (*p* *=*0.024) were more than those in neighboring tissues. The positive rate of FOXM1 in ESCC (*p* < 0.001) was greater than that in LIN. The above results demonstrated that UBE2S, HIF‐1α, and FOXM1 expression may be related to the ESCC development.

Figure 7UBE2S, HIF‐1α, and FOXM1 expression in esophageal intraepithelial neoplasia and normal mucosa tissue. (A) Reduced expression of UBE2S in normal mucosa tissue. (B, C) Elevated expression of UBE2S in HIN and LIN. (D) Low HIF‐1α expression in normal mucosa tissue. (E, F) Elevated HIF‐1α expression in HIN and LIN. (G) Reduced FOXM1 expression in normal mucosa tissue. (H, I) High expression of FOXM1 in HIN and LIN (magnification, ×100 in magnified window; scale bar: 200 *μ*m).

**Figure:**
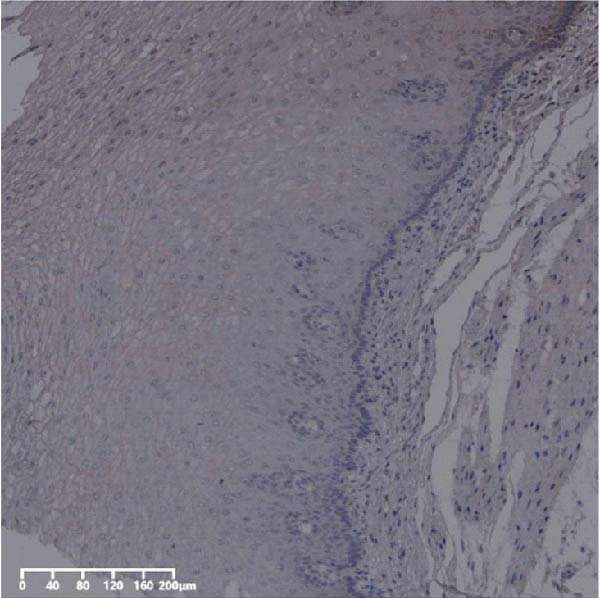
(A)

**Figure:**
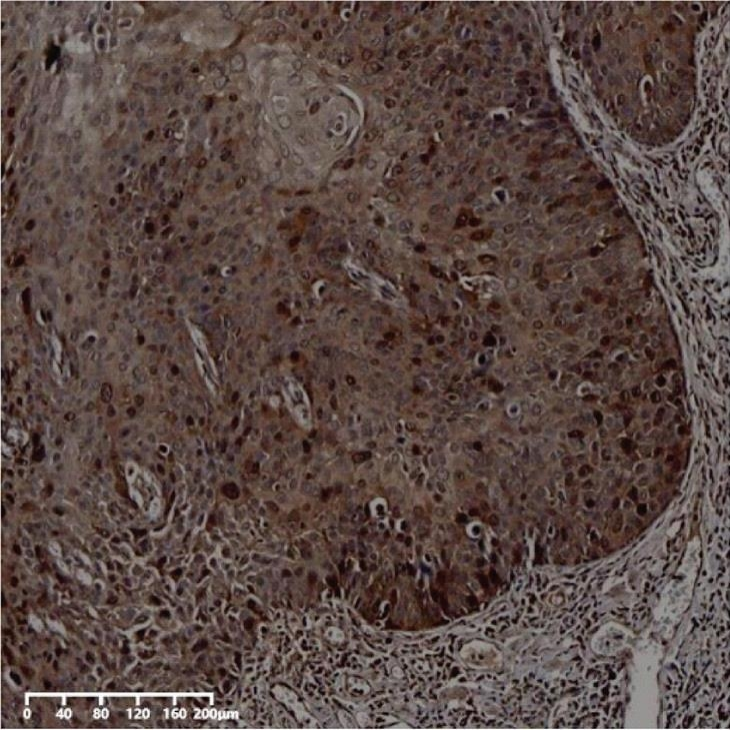
(B)

**Figure:**
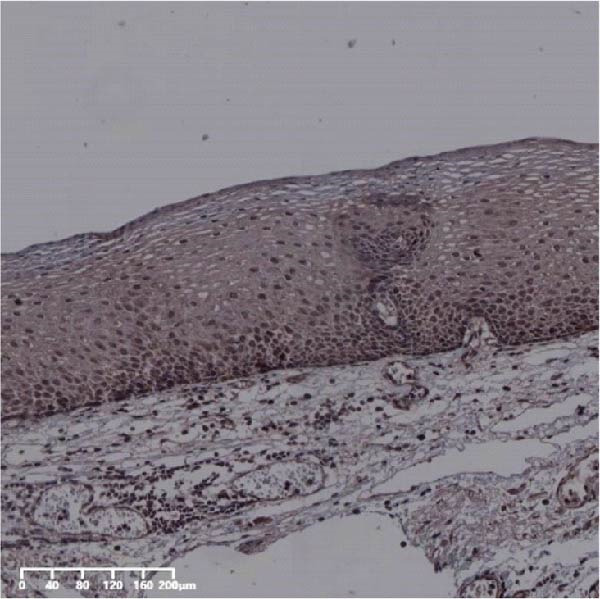
(C)

**Figure:**
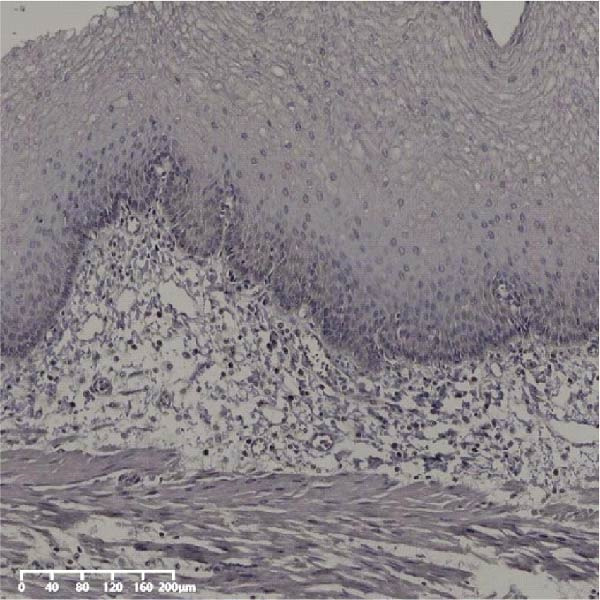
(D)

**Figure:**
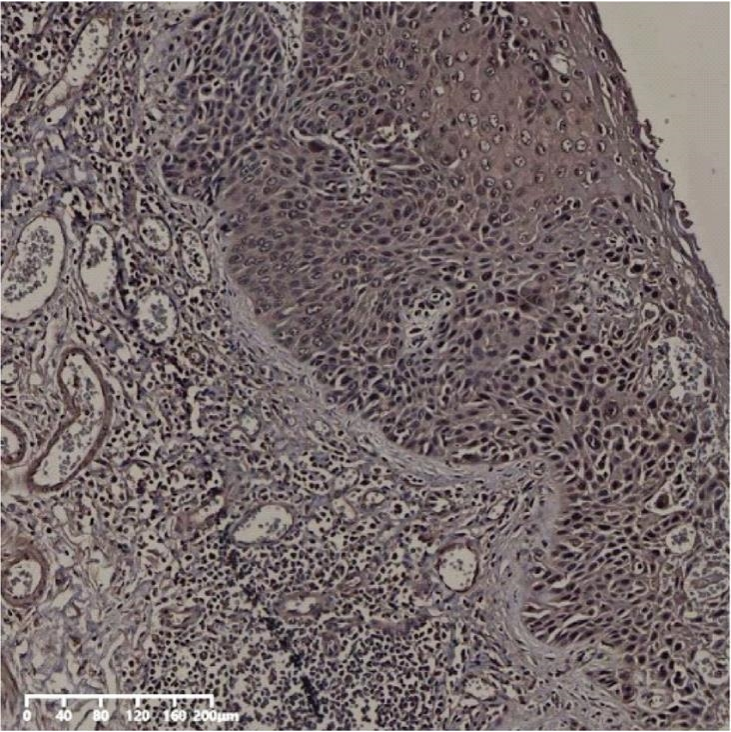
(E)

**Figure:**
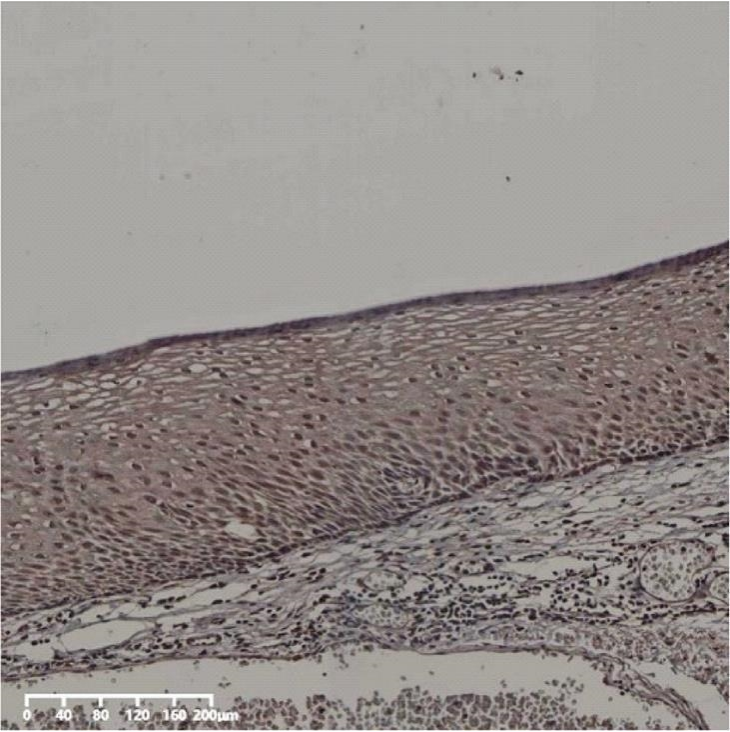
(F)

**Figure:**
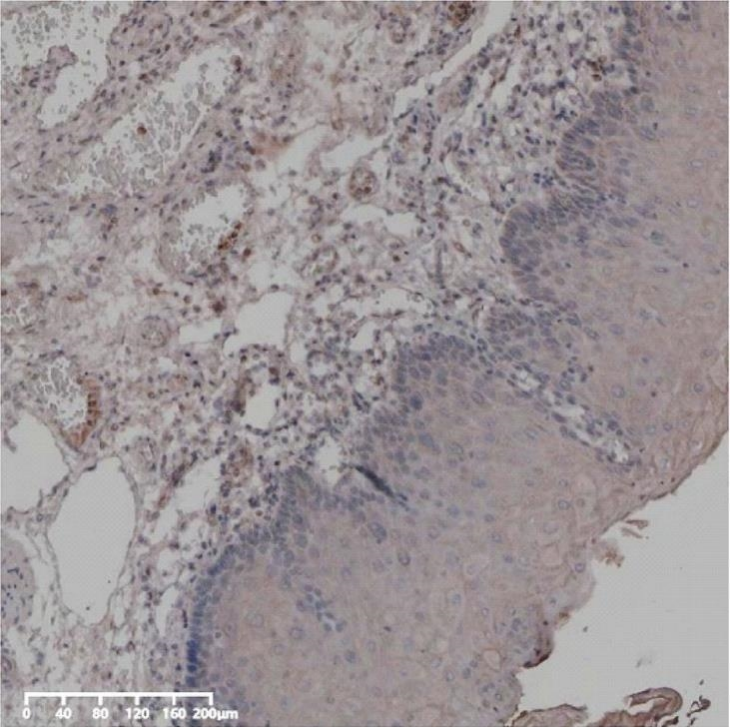
(G)

**Figure:**
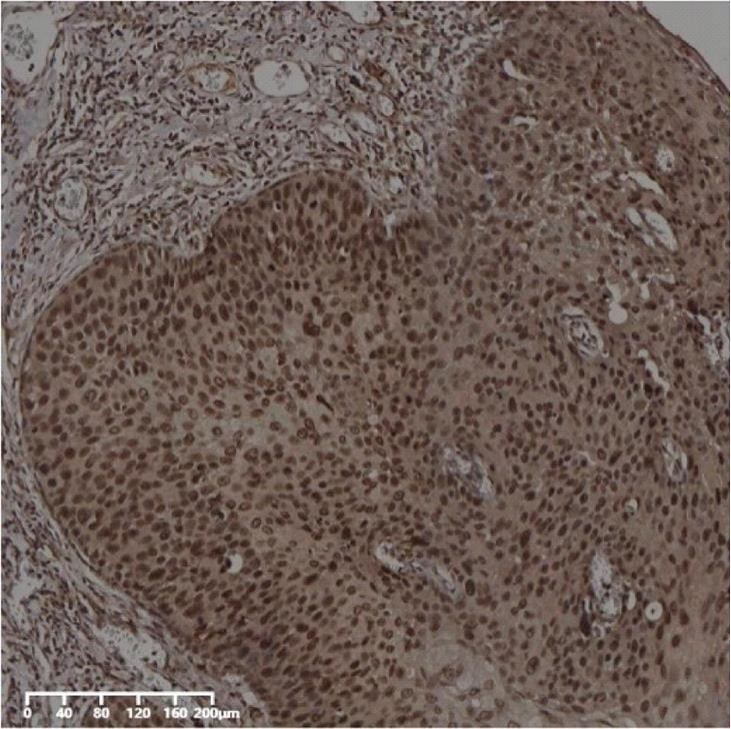
(H)

**Figure:**
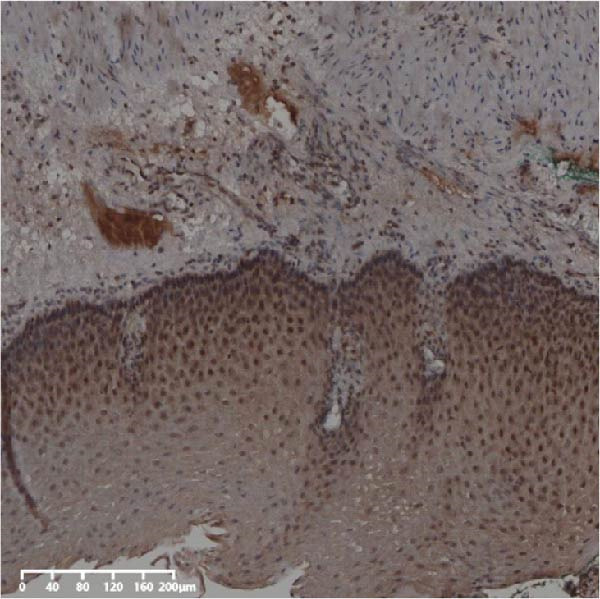
(I)

**Table 6:** The positive expression rate of UBE2S, HIF‐1α, and FOXM1 in different classification.

Classification	*n*	UBE2S			HIF‐1α			FOXM1		
		Low expression	High expression	*p*‐Value	Low expression	High expression	*p*‐Value	Low expression	High expression	*p*‐Value
				<0.001			<0.001			<0.001
Normal mucosa tissue	49	38 (77.6)	11 (22.4)	^a,b^	42 (85.7)	7 (14.3)	^e,f^	40 (81.6)	9 (18.4)	^i,j^
LIN	49	27 (55.1)	22 (44.9)	^c,d^	34 (69.4)	15 (30.6)	^g,h^	37 (75.5)	12 (24.5)	^k^
HIN	49	13 (26.5)	36 (73.5)		19 (38.8)	30 (61.2)		26 (53.1)	23 (46.9)	
ESCC	49	12 (24.5)	37 (75.5)		18 (36.7)	31 (63.3)		14 (28.6)	35 (71.4)	

^a^ *p* < 0.001 (normal vs. HIN).

^b^ *p* < 0.001 (normal vs. ESCC).

^c^ *p* = 0.028 (LIN vs. HIN).

^d^ *p* = 0.015 (LIN vs. ESCC).

^e^ *p* < 0.001 (normal vs. HIN).

^f^ *p* < 0.001 (normal vs. ESCC).

^g^ *p* = 0.013 (LIN vs. HIN).

^h^ *p* = 0.007 (LIN vs. ESCC).

^i^ *p* = 0.024 (normal vs. HIN).

^j^ *p* < 0.001 (normal vs. ESCC).

^k^ *p* < 0.001 (LIN vs. ESCC).

### 3.9. Connection of UBE2S, HIF‐1α, and FOXM1 Expression in Esophageal Intraepithelial Neoplasia

Spearman correlation analysis was conducted on the UBE2S, HIF‐1α, and FOXM1 expression in 49 patients of LIN and 49 individuals of HIN. The results meant that in HIN (*r* = 0.565, *p* < 0.001) and LIN (*r* = 0.291, *p* = 0.043), the expressions of UBE2S and HIF‐1α were positively connected. In HIN (*r* = 0.473, *p* = 0.001) and LIN (*r* = 0.440, *p* = 0.002), the expressions of UBE2S and FOXM1 were positively correlated. In HIN (*r* = 0.497, *p* < 0.001) and LIN (*r* = 0.343, *p* = 0.016), the expressions of HIF‐1α and FOXM1 were positively connected (Tables 7–10). To sum up, UBE2S, HIF‐1α, and FOXM1 expression elevated with the elevation of tumor grade, and the correlation coefficient increased.

**Table 7:** Correlation between UBE2S, HIF‐1α, and FOXM1 in HIN tissues.

Protein	UBE2S expression	
	*r*	*p*‐Value
HIF‐1α expression	0.565	<0.001
FOXM1 expression	0.473	0.001

*Note:* The Spearman correlation analysis was utilized to test the connection between UBE2S, HIF‐1α, and FOXM1 in HIN.

**Table 8:** Correlation between HIF‐1α and FOXM1 in HIN tissues.

Protein	HIF‐1α expression	
	*r*	*p*‐Value
FOXM1 expression	0.497	<0.001

*Note:* The Spearman correlation analysis was utilized to test the connection between HIF‐1α and FOXM1 in HIN.

**Table 9:** Correlation between UBE2S, HIF‐1α, and FOXM1 in LIN tissues.

Protein	UBE2S expression	
	*r*	*p*‐Value
HIF‐1α expression	0.291	0.043
FOXM1 expression	0.440	0.002

*Note:* The Spearman correlation analysis was utilized to test the connection between UBE2S, HIF‐1α, and FOXM1 in LIN.

**Table 10:** Correlation between HIF‐1α and FOXM1 in LIN tissues.

Protein	HIF‐1α expression	
	*r*	*p*‐Value
FOXM1 expression	0.343	0.016

*Note:*The Spearman correlation analysis was utilized to test the connection between HIF‐1α and FOXM1 in LIN.

## 4. Discussion

China has the greatest prevalence of ESCC, representing about 57% of the total worldwide cases [26]. Xinjiang is a region in China with an increased prevalence of ESCC, and the majority of patients are diagnosed in the advanced disease stages. While surgical intervention is the primary approach for treating EC, it has poor long‐term efficacy, resulting in a low quality of life for postoperative patients and a 5‐year survival rate of fewer than 20% [3]. In recent years, molecular targeted drugs and immunotherapy have made some progress in neoadjuvant therapy for cancer of the esophagus, but there is still an absence of effective strategies. Discovering novel molecular therapeutic targets for additional treatment for individuals with esophageal cancer has immense importance.

UBE2S, a member of the enzyme family ubiquitin‐conjugating, is a ubiquitin ligase regulated by the cell cycle. As an important factor in promoting the anaphase‐promoting complex (APC/C), UBE2S extends ubiquitin chains on APC/C substrates to promote mitotic exit [8]. UBE2S was overexpressed in many tumors, such as ESCC [4], oral squamous cell cancer [27], gastric malignancy [28], and breast cancer [29]. It had a significant impact on the initiation, progression, reappearance, and metastasis of malignancies. UBE2S expression level in cervical cancer was demonstrated to have a positive association with the AJCC stage [18]. In carcinoma of the oral squamous cell, UBE2S expression was positively connected with the primary tumor size [27]. In ESCC, the elevated UBE2S expression was connected to the adverse reaction of neoadjuvant therapy and the worse survival rate [4]. Previous research has shown that DNA methylation can exert a negative modulation on UBE2S expression in ovarian cancer, rectal adenocarcinoma, testicular germ cell tumor (TGCT), and so on. For instance, significantly decreased UBE2S expression was regulated by DNA methylation patterns of different loci in TGCT via probes cg26692860, cg24973886, cg23437684, and cg23373897. Furthermore, consistent trends between UBE2S and RNA modification related genes (including m1A, m5C, and m6A) were also observed in numerous tumors such as esophageal carcinoma, adenoid cystic carcinoma, and ovarian cancer [30]. In our study, there were no significant differences in the expression of UBE2S between normal tissues from Han and Kazakh. The increased UBE2S expression was linked to ethnicity, which may be connected to the DNA methylation and RNA modification among Kazakh patients with ESCC, the specific content requires further investigation. Our current results showed that UBE2S overexpression was related to the tumor location, however, with only nine samples in the upper position, we need to expand the sample size to further verify. The prognosis of subjects with elevated UBE2S expression was poor. From the results, no statistical significance was detected between the UBE2S expression and lymph node metastasis, differentiation degree, and AJCC. While UBE2S expression may still have a connection with the ESCC progression, it is possible that this finding is influenced by the variability of the specimens.

The investigation has demonstrated that UBE2S forms connections with pVHL and directs it toward ubiquitin‐mediated proteolysis in cells, resulting in the stabilization of HIF‐1α. UBE2S is identified simultaneously with HIF‐1α in human primary hepatic, colon, and breast cancers, metastatic cholangiocarcinoma, and cells of colon cancer. There is an inverse association between the amount of UBE2S and the level of pVHL in most cancer cell lines. Forced UBE2S expression in vitro and in vivo enhances the growth, invasion, and metastasis of malignant cells by affecting the pathway of pVHL–HIF [17]. UBE2S had the ability to raise the EMT in pancreatic ductal adenocarcinoma. This was done via the pathway of VHL/HIF‐1α/STAT3, employing the UPS [12]. Investigations demonstrated a connection between the rise of UBE2S and the reduction of VHL, in addition to the elevated levels of HIF‐1α, in cervical malignancies and ESCC [4, 31]. In ESCC cell culture, the increased UBE2S was associated with higher expression of HIF‐1α and VEGF, while the level of VHL was decreased [4]. We conducted a study on the positive connection between UBE2S and HIF‐1α expression in ESCC, which aligned with earlier research findings.

Under hypoxia, the N‐terminal trans‐activation domain has a nuclear localization signal, which helps HIF‐1α to combine with nucleoporin and merge into the nucleus, and forms a stable heterodimer with *β* subunit located in the nucleus. As a result, the hydroxylation is damaged, and HIF‐1α is stable [32, 33]. Due to the rapid proliferation of malignant tumors, malignant tumor cells are in a state of hypoxia for a long time. HIF‐1α specifically modifies the expression of related tumor genes after translation to adapt to the hypoxic environment, like breast [34], colon [35], and small cell lung cancers [14], and HIF‐1α expression increases. When cancer cells are subjected to hypoxia, HIF‐1α accumulates in the nucleus and binds to related target genes. The combined target genes are related to the growth, metastasis, and invasion of malignant tumor cells and the medication resistance of tumor cells [33]. Xia et al. [36] discovered that HIF‐1 has the ability to attach itself to the HIF‐1 binding location in the FOXM1 promoter. This interaction led to an increase in the FOXM1 expression, which in turn enhanced the growth, migration, and invasion of cancerous cells [24, 36]. Our team mainly studied the expression relationship of UBE2S, HIF‐1α, and FOXM1 in ESCC and HIN and LIN. Utilizing Spearman correlation analysis, we observed a positive link between UBE2S, HIF‐1α, and FOXM1 in cases of ESCC, HIN, and LIN. Notably, in HIN and LIN, the coefficient correlation of UBE2S, HIF‐1α, and FOXM1 increased as an increase in tumor grade. What is more, in our investigation, HIF‐1α did not directly impact the OS and PFS in ESCC, but the prognosis of patients coexpressing UBE2S, HIF‐1α, and FOXM1 was the worst in ESCC. From the available information, it is inferred that UBE2S/HIF‐1α may affect the progress and prognosis of ESCC by regulating the expression of FOXM1.

Furthermore, our investigation revealed that UBE2S, HIF‐1α, and FOXM1 exhibited significant upregulation in both esophageal intraepithelial neoplasia and cancer, with statistically significant variations observed. UBE2S, HIF‐1α, and FOXM1 may play a role in the progression of esophageal HIN and LIN to cancer. UBE2S, HIF‐1α, and FOXM1 were all positively correlated, which may affect the prognosis of ESCC. UBE2S, HIF‐1α, and FOXM1 have the potential to serve as precise markers for the assessment and prognosis of individuals at elevated risk for malignancy of the esophagus.

In cancer of the ovaries, UBE2S increased the growth and Olaparib resistance of tumors in its enzymatic activity‐dependent technique [37]. The expression of UBE2S had a strong connection with the malignancy of glioma and its resistance to chemoradiotherapy. Additionally, it served as a vital marker indicating an unfavorable prognosis [11]. Through in vitro research, overexpression of UBE2S in HCC cells caused a rise in resistance to 5‐fluorouracil (5‐FU) and oxaliplatin, while when UBE2S was suppressed, a reverse impact was observed [38]. Overexpression of HIF‐1 α in glioblastoma cells [39], hepatocellular carcinoma [40], and ovarian cancer [41] may be insensitive to radiation or resistant to chemotherapy. In cervical cancer [42], prostate cancer [43], and nasopharyngeal carcinoma [44], FOXM1 caused paclitaxel resistance by modulating the expression of ATP‐binding cassette subfamily C member 5 (ABCC5), in vitro and in vivo studies demonstrate that FOXM1‐specific inhibitors significantly weaken paclitaxel resistance. In this investigation, it was worth noting that UBE2S negative patients with radiotherapy and chemotherapy had significantly prolonged OS and PFS; That was, overexpression of UBE2S may reduce the patient’s sensitivity to chemotherapy and radiotherapy. HIF‐1α and FOXM1 came to the same conclusion as UBE2S. To sum up, UBE2S, HIF‐1α, and FOXM1 were highly expressed in ESCC, and it was easy to develop drug resistance to postoperative treatment. Therefore, their inhibition may enhance the efficacy of postoperative treatment and improve the prognosis of patients.

This investigation has some restrictions. At present, UBE2S, HIF‐1α, and FOXM1 function in ESCC, and their influence on prognosis have only been preliminarily verified. Immunohistochemical analysis can not determine its mechanism, and it needs to be further analyzed at the cellular and animal levels.

## 5. Conclusions

The expressions of UBE2S, HIF‐1α, and FOXM1 in ESCC, HIN, LIN, and healthy esophageal mucosa were different, suggesting that UBE2S, HIF‐1α, and FOXM1 may be related to the development of ESCC. The raised UBE2S expression was correlated with ethnicity. The high HIF‐1α and FOXM1 expression was strictly correlated with metastasis of the lymph node. UBE2S, HIF‐1α, and FOXM1 were positively correlated in ESCC and HIN, LIN. The higher the grade of neoplasia, the greater the correlation coefficient. The coexpression of UBE2S, HIF‐1α, and FOXM1 affected the prognosis of patients. Therefore, UBE2S, HIF‐1α, and FOXM1 can be used as molecular markers for screening and prognosis of ESCC. Additional predictive biomarkers for malignancy are required. The aforementioned findings offer a theoretical foundation for potential therapeutic objectives in the treatment of ESCC.

## Ethics Statement

This study was approved by the Ethics Committee of the First Affiliated Hospital of Xinjiang Medical University (20180223‐08) to conduct experimental research using clinicopathological specimens from patients with esophageal squamous cell carcinoma.

## Disclosure

The finalized paper was reviewed and approved by all the authors. Every contributor has verified and validated the authenticity of the original data. All the authors provided their final acceptance for the published edition.

## Conflicts of Interest

The authors declare no conflicts of interest.

## Author Contributions

Wan Li, Mengyan Li, and Yuqing Ma conceived and designed the study. Wan Li and Liping Su performed the experiments. Wan Li performed the data analysis and wrote the article. Wan Li and Xianwen Liu were responsible for the data interpretation. Yuanyuan Lv did the bioinformatics analysis of TCGA database. Wan Li and Keming Zhou contributed equally to this study.

## Funding

This work was supported by the State Key Laboratory of Pathogenesis and Prevention of High Incidence in Central Asia, Jointly Established by Provincial and Ministry (Grants SKL‐HIDCA‐2022‐10 and SKL‐HIDCA‐2020‐4), the Natural Science Foundation of Xinjiang Uygur Autonomous Region (Grant 2022D01D71), and the National Natural Science Foundation of China (Grant 81860422).

## Supporting Information

Additional supporting information can be found online in the Supporting Information section.

## Supporting information


**Supporting Information** Table S1. General features of patients with ESCC.

## Data Availability

The datasets utilized and/or examined in the present investigation may be obtained from the corresponding author upon an adequate demand.

## References

[1] Cao W. , Chen H.-D. , Yu Y.-W. , Li N. , and Chen W.-Q. , Changing Profiles of Cancer Burden Worldwide and in China: A Secondary Analysis of the Global Cancer Statistics 2020, Chinese Medical Journal. (2021) 134, no. 7, 783–791, 10.1097/CM9.0000000000001474. 33734139 PMC8104205

[2] Yang J. , Dou Z. , and Peng X. , et al.Transcriptomics and Proteomics Analyses of Anti-Cancer Mechanisms of TR35-An Active Fraction From Xinjiang Bactrian Camel Milk in Esophageal Carcinoma Cell, Clinical Nutrition. (2019) 38, no. 5, 2349–2359, 10.1016/j.clnu.2018.10.013, 2-s2.0-85072561709. 30420292

[3] Li H. , Sun D. , and Cao M. , et al.Risk Prediction Models for Esophageal Cancer: A Systematic Review and Critical Appraisal, Cancer Medicine. (2021) 10, no. 20, 7265–7276, 10.1002/cam4.4226. 34414682 PMC8525074

[4] Chen M. F. , Lee K. D. , and Lu M. S. , et al.The Predictive Role of E2-EPF Ubiquitin Carrier Protein in Esophageal Squamous Cell Carcinoma, Journal of Molecular Medicine. (2009) 87, no. 3, 307–320, 10.1007/s00109-008-0430-3, 2-s2.0-60549107673. 19083192

[5] Li X. , Elmira E. , Rohondia S. , Wang J. , Liu J. , and Dou Q. P. , A Patent Review of the Ubiquitin Ligase System: 2015-2018, Expert Opinion on Therapeutic Patents. (2018) 28, no. 12, 919–937, 10.1080/13543776.2018.1549229, 2-s2.0-85057265485. 30449221 PMC6398165

[6] Liu Z. and Xu L. , UBE2S Promotes the Proliferation and Survival of Human Lung Adenocarcinoma Cells, BMB Reports. (2018) 51, no. 12, 642–647, 10.5483/BMBRep.2018.51.12.138, 2-s2.0-85059232752. 30545437 PMC6330942

[7] Chang L. , Zhang Z. , Yang J. , McLaughlin S. H. , and Barford D. , Atomic Structure of the APC/C and its Mechanism of Protein Ubiquitination, Nature. (2015) 522, no. 7557, 450–454, 10.1038/nature14471, 2-s2.0-84933038158. 26083744 PMC4608048

[8] Garnett M. J. , Mansfeld J. , and Godwin C. , et al.UBE2S Elongates Ubiquitin Chains on APC/C Substrates to Promote Mitotic Exit, Nature Cell Biology. (2009) 11, no. 11, 1363–1369, 10.1038/ncb1983, 2-s2.0-70449529843. 19820702 PMC2875106

[9] Zhang M. , Liu Y. , and Yin Y. , et al.UBE2S Promotes the Development of Ovarian Cancer by Promoting PI3K/AKT/mTOR Signaling Pathway to Regulate Cell Cycle and Apoptosis, Molecular Medicine. (2022) 28, no. 1, 10.1186/s10020-022-00489-2, 62. 35658829 PMC9166599

[10] Peng S. , Chen X. , and Huang C. , et al.UBE2S as a Novel Ubiquitinated Regulator of p16 and *β*-Catenin to Promote Bone Metastasis of Prostate Cancer, International Journal of Biological Sciences. (2022) 18, no. 8, 3528–3543, 10.7150/ijbs.72629. 35637955 PMC9134922

[11] Hu L. , Cheng X. , and Binder Z. , et al.Molecular and Clinical Characterization of UBE2S in Glioma as a Biomarker for Poor Prognosis and Resistance to Chemo-Radiotherapy, Frontiers in Oncology. (2021) 11, 10.3389/fonc.2021.640910, 640910. 34123793 PMC8190380

[12] Wang L. , Liang Y. , and Li P. , et al.Oncogenic Activities Of UBE2S Mediated By VHL/HIF-1alpha/STAT3 Signal Via the Ubiquitin-Proteasome System In PDAC, OncoTargets and Therapy. (2019) 12, 9767–9781, 10.2147/OTT.S228522. 31814735 PMC6863183

[13] Zhang W. , Yuan W. , Song J. , Wang S. , and Gu X. , LncRNA CPS1-IT1 Suppresses EMT and Metastasis of Colorectal Cancer by Inhibiting Hypoxia-Induced Autophagy Through Inactivation of HIF-1α, Biochimie. (2018) 144, 21–27, 10.1016/j.biochi.2017.10.002, 2-s2.0-85031826384. 29017924

[14] Lin C.-S. , Liu T.-C. , Lee M.-T. , Yang S.-F. , and Tsao T. C.-Y. , Independent Prognostic Value of Hypoxia-Inducible Factor 1-Alpha Expression in Small Cell Lung Cancer, International Journal of Medical Sciences. (2017) 14, no. 8, 785–790, 10.7150/ijms.19512, 2-s2.0-85026813621. 28824314 PMC5562133

[15] Daponte A. , Ioannou M. , and Mylonis I. , et al.Prognostic Significance of Hypoxia-Inducible Factor 1 Alpha (HIF-1 Alpha) Expression in Serous Ovarian Cancer: An Immunohistochemical Study, BMC Cancer. (2008) 8, no. 1, 10.1186/1471-2407-8-335, 2-s2.0-62349084794, 335. 19014607 PMC2651893

[16] Liang X. , Zheng M. , Jiang J. , Zhu G. , Yang J. , and Tang Y. , Hypoxia-Inducible Factor-1 Alpha, in Association With TWIST2 and SNIP1, is a Critical Prognostic Factor in Patients With Tongue Squamous Cell Carcinoma, Oral Oncology. (2011) 47, no. 2, 92–97, 10.1016/j.oraloncology.2010.11.014, 2-s2.0-79551685392. 21167768

[17] Jung C. R. , Hwang K. S. , and Yoo J. , et al.E2-EPF UCP Targets pVHL for Degradation and Associates With Tumor Growth and Metastasis, Nature Medicine. (2006) 12, no. 7, 809–816, 10.1038/nm1440, 2-s2.0-33745901606. 16819549

[18] Liang J. , Nishi H. , and Bian M. L. , et al.The Ubiquitin-Conjugating Enzyme E2-EPF Is Overexpressed in Cervical Cancer and Associates With Tumor Growth, Oncology Reports. (2012) 28, no. 4, 1519–1525, 10.3892/or.2012.1949, 2-s2.0-84866456621. 22895574

[19] Wierstra I. , FOXM1 (Forkhead Box M1) in Tumorigenesis: Overexpression in Human Cancer, Implication in Tumorigenesis, Oncogenic Functions, Tumor-Suppressive Properties, and Target of Anticancer Therapy, Advanced Cancer Research. (2013) 119, 191–419. 10.1016/B978-0-12-407190-2.00016-2 23870513

[20] Halasi M. and Gartel A. L. , FOX(M1) News--it is Cancer, Molecular Cancer Therapeutics. (2013) 12, no. 3, 245–254. 23443798 10.1158/1535-7163.MCT-12-0712 PMC3596487

[21] Yang C. , Chen H. , and Tan G. , et al.FOXM1 Promotes the Epithelial to Mesenchymal Transition by Stimulating the Transcription of Slug in Human Breast Cancer, Cancer Letters. (2013) 340, no. 1, 104–112, 10.1016/j.canlet.2013.07.004, 2-s2.0-84883763813. 23856032

[22] Mariette C. , Piessen G. , and Triboulet J.-P. , Therapeutic Strategies in Oesophageal Carcinoma: Role of Surgery and Other Modalities, The Lancet Oncology. (2007) 8, no. 6, 545–553, 10.1016/S1470-2045(07)70172-9, 2-s2.0-34249278457. 17540306

[23] Higurashi M. , Maruyama T. , and Nogami Y. , et al.High Expression of FOXM1 Critical for Sustaining Cell Proliferation in Mitochondrial DNA-less Liver Cancer Cells, Experimental Cell Research. (2020) 389, no. 1, 10.1016/j.yexcr.2020.111889, 111889. 32032602

[24] Tang C. , Liu T. , and Wang K. , et al.Transcriptional Regulation of FOXM1 by HIF-1α Mediates Hypoxia-Induced EMT in Prostate Cancer, Oncology Reports. (2019) 42, no. 4, 1307–1318, 10.3892/or.2019.7248, 2-s2.0-85071007613. 31364741 PMC6718104

[25] Kuai X.-Y. , Lei Z.-Y. , Liu X.-S. , and Shao X.-Y. , The Interaction of GLUT1 and FOXM1 Leads to a Poor Prognosis in Colorectal Cancer, Anti-Cancer Agents in Medicinal Chemistry. (2020) 20, no. 8, 941–950, 10.2174/1871520620666200318094618. 32188390

[26] Morgan E. , Soerjomataram I. , and Rumgay H. , et al.The Global Landscape of Esophageal Squamous Cell Carcinoma and Esophageal Adenocarcinoma Incidence and Mortality in 2020 and Projections to 2040: New Estimates From GLOBOCAN 2020, Gastroenterology. (2022) 163, no. 3, 649–658.e2, 10.1053/j.gastro.2022.05.054. 35671803

[27] Yoshimura S. , Kasamatsu A. , and Nakashima D. , et al.UBE2S Associated With OSCC Proliferation by Promotion of P21 Degradation via the Ubiquitin-Proteasome System, Biochemical and Biophysical Research Communications. (2017) 485, no. 4, 820–825, 10.1016/j.bbrc.2017.02.138, 2-s2.0-85014387988. 28257844

[28] Zhao R. , Yu Z. , Mao X. , Zheng Y. , Wang Y. , and Zhou Y. , Knockout of UBE2S Inhibits the Proliferation of Gastric Cancer Cells and Induces Apoptosis by FAS-Mediated Death Receptor Pathway, Experimental Cell Research. (2022) 419, no. 1, 10.1016/j.yexcr.2022.113293, 113293. 35863455

[29] Lin C. Y. , Yu C. J. , and Liu C. Y. , et al.CDK4/6 Inhibitors Downregulate the Ubiquitin-Conjugating Enzymes UBE2C/S/T Involved in the Ubiquitin-Proteasome Pathway in ER + Breast Cancer, Clinical and Translational Oncology. (2022) 24, no. 11, 2120–2135, 10.1007/s12094-022-02881-0. 35917055

[30] Bao H. , Luo Y. , Ding G. , and Fu Z. , A Pan-Cancer Analysis of UBE2S in Tumorigenesis, Prognosis, Pathway, Immune Infiltration and Evasion, and Therapy Response From an Immune-Oncology Perspective, Journal of Oncology. (2022) 2022, 10.1155/2022/3982539, 3982539. 35578600 PMC9107357

[31] Lin T. H. , Hsu W. H. , and Tsai P. H. , et al.Dietary Flavonoids, Luteolin and Quercetin, Inhibit Invasion of Cervical Cancer by Reduction of UBE2S Through Epithelial-Mesenchymal Transition Signaling, Food & Function. (2017) 8, no. 4, 1558–1568, 10.1039/C6FO00551A, 2-s2.0-85018739590. 28277581

[32] Zhang Q. , Han Z. , Zhu Y. , Chen J. , and Li W. , Role of Hypoxia Inducible Factor-1 in Cancer Stem Cells (Review), Molecular Medicine Reports. (2021) 23, no. 1. 10.3892/mmr.2020.11655 PMC7673349 33179080

[33] Tong W. W. , Tong G. H. , and Liu Y. , Cancer Stem Cells and Hypoxia-Inducible Factors (Review), International Journal of Oncology. (2018) 53, no. 2, 469–476. 29845228 10.3892/ijo.2018.4417

[34] Badowska-Kozakiewicz A. M. , Sobol M. , and Patera J. , Expression of Multidrug Resistance Protein P-Glycoprotein in Correlation With Markers of Hypoxia (HIF-1α, EPO, EPO-R) in Invasive Breast Cancer With Metastasis to Lymph Nodes, Archives of Medical Science. (2017) 13, no. 6, 1303–1314, 10.5114/aoms.2016.62723, 2-s2.0-85032688481. 29181060 PMC5701689

[35] Rodríguez M. E. , Catrinacio C. , Ropolo A. , Rivarola V. A. , and Vaccaro M. I. , A Novel HIF-1α /VMP1-Autophagic Pathway Induces Resistance to Photodynamic Therapy in Colon Cancer Cells, Photochemical & Photobiological Sciences. (2017) 16, no. 11, 1631–1642, 10.1039/c7pp00161d, 2-s2.0-85033572468. 28936522

[36] Xia L. M. , Huang W. J. , and Wang B. , et al.Transcriptional up-Regulation of FOXM1 in Response to Hypoxia is Mediated by HIF-1, Journal of Cellular Biochemistry. (2009) 106, no. 2, 247–256, 10.1002/jcb.21996, 2-s2.0-61449153846. 19097132

[37] Hu W. , Li M. , Chen Y. , and Gu X. , UBE2S Promotes the Progression and Olaparib Resistance of Ovarian Cancer Through Wnt/*β*-Catenin Signaling Pathway, Journal of Ovarian Research. (2021) 14, no. 1, 10.1186/s13048-021-00877-y, 121. 34535173 PMC8447717

[38] Gui L. , Zhang S. , Xu Y. , Zhang H. , Zhu Y. , and Kong L. , UBE2S Promotes Cell Chemoresistance Through PTEN-AKT Signaling in Hepatocellular Carcinoma, Cell Death Discovery. (2021) 7, no. 1, 10.1038/s41420-021-00750-3, 357. 34785642 PMC8595659

[39] Ahmed E. M. , Bandopadhyay G. , Coyle B. , and Grabowska A. , A HIF-Independent, CD133-Mediated Mechanism of Cisplatin Resistance in Glioblastoma Cells, Cellular Oncology. (2018) 41, no. 3, 319–328, 10.1007/s13402-018-0374-8, 2-s2.0-85042627695. 29492900 PMC5951876

[40] Chen Y. , Li H. , and Chen D. , et al.Hypoxic Hepatocellular Carcinoma Cells Acquire Arsenic Trioxide Resistance by Upregulating HIF-1α Expression, Digestive Diseases and Sciences. (2022) 67, no. 8, 3806–3816, 10.1007/s10620-021-07202-z. 34383201

[41] Ai Z. , Lu Y. , Qiu S. , and Fan Z. , Overcoming Cisplatin Resistance of Ovarian Cancer Cells by Targeting HIF-1-Regulated Cancer Metabolism, Cancer Letters. (2016) 373, no. 1, 36–44, 10.1016/j.canlet.2016.01.009, 2-s2.0-84958074679. 26801746 PMC4769873

[42] Hou Y. , Dong Z. , and Zhong W. , et al.FOXM1 Promotes Drug Resistance in Cervical Cancer Cells by Regulating ABCC5 Gene Transcription, BioMed Research International. (2022) 2022, no. 1, 10.1155/2022/3032590, 3032590. 35141332 PMC8820921

[43] Yu H. , Xu Z. , and Guo M. , et al.FOXM1 Modulates Docetaxel Resistance in Prostate Cancer by Regulating KIF20A, Cancer Cell International. (2020) 20, no. 1, 10.1186/s12935-020-01631-y, 545. 33292277 PMC7653758

[44] Hou Y. , Zhu Q. , and Li Z. , et al.The FOXM1-ABCC5 Axis Contributes to Paclitaxel Resistance in Nasopharyngeal Carcinoma Cells, Cell Death & Disease. (2017) 8, no. 3, 10.1038/cddis.2017.53, 2-s2.0-85026612452, e2659. 28277541 PMC5386553

