# Supplementary material for: Prognostic Value of UBE2S, HIF‐1α, and FOXM1 Expression in Esophageal Squamous Cell Carcinoma
Source: Anal Cell Pathol (Amst). 2025 Dec 20;2025:3557238. doi: 10.1155/ancp/3557238 (PMC12717677; doi:10.1155/ancp/3557238)
Supplement: Supplementary file 1 — Supporting Information Table S1. General features of patients with ESCC. [file ANCP-2025-3557238-s001.pdf]

Supplemental table 1. General features of patients with ESCC.

Clinical parameters	n(%)
Age	
<60	59(34.1)
≥60	114(65.9)
Gender	
Male	120(69.4)
Female	53(30.6)
Ethnicity	
Han	90(52.0)
Kazakh	83(48.0)
Tumor location	
Upper	9(5.2)
Middle	103(59.5)
Lower	61(35.3)
Tumor size(cm)	
＜3	55(31.8)
≥3	118(68.2)
Differentiation	
Poor	27(15.6)
Moderate	97(56.1)
Well	49(28.3)
Lymph metastasis	
No	122(70.5)
Yes	51(29.5)
Invasive depth	
Mucosa	3(1.7)
Muscularis	72(41.6)
Full thickness	98(56.7)
AJCC stage	
I+II	127(73.4)
III+IV	46(26.6)
Vascular invasion	
No	143(82.7)
Yes	30(17.3)
Nerve invasion	
No	143(82.7)
Yes	30(17.3)
Hematogenous metastasis	
No	149(86.1)
Yes	24(13.9)
Postoperative treatment	
No	95(54.9)
Yes	78(45.1)

Patients (n=173)
